# Role of riboflavin biosynthesis gene duplication and transporter in *Aeromonas salmonicida* virulence in marine teleost fish

**DOI:** 10.1080/21505594.2023.2187025

**Published:** 2023-03-09

**Authors:** Hajarooba Gnanagobal, Trung Cao, Ahmed Hossain, Ignacio Vasquez, Setu Chakraborty, Joy Chukwu-Osazuwa, Danny Boyce, María Jesus Espinoza, Víctor Antonio García-Angulo, Javier Santander

**Affiliations:** a Marine Microbial Pathogenesis and Vaccinology Laboratory, Department of Ocean Sciences, Memorial University of Newfoundland, St John’s, Canada; b The Dr. Joe Brown Aquatic Research Building (JBARB), Ocean Sciences Centre, Memorial University of Newfoundland, St John’s, Canada; c Microbiology and Mycology Program, Institute of Biomedical Sciences, Facultad de Medicina, Universidad de Chile, Santiago, Chile

**Keywords:** Riboflavin biosynthesis, riboflavin transport, gene duplication, *Aeromonas salmonicida* virulence

## Abstract

Active flavins derived from riboflavin (vitamin B_2_) are essential for life. Bacteria biosynthesize riboflavin or scavenge it through uptake systems, and both mechanisms may be present. Because of riboflavin’s critical importance, the redundancy of riboflavin biosynthetic pathway (RBP) genes might be present. *Aeromonas salmonicida*, the aetiological agent of furunculosis, is a pathogen of freshwater and marine fish, and its riboflavin pathways have not been studied. This study characterized the *A. salmonicida* riboflavin provision pathways. Homology search and transcriptional orchestration analysis showed that *A. salmonicida* has a main riboflavin biosynthetic operon that includes *ribD*, *ribE1*, *ribBA*, and *ribH* genes. Outside the main operon, putative duplicated genes *ribA*, *ribB* and *ribE*, and a *ribN* riboflavin importer encoding gene, were found. Monocistronic mRNA *ribA*, *ribB* and *ribE2* encode for their corresponding functional riboflavin biosynthetic enzyme. While the product of *ribBA* conserved the RibB function, it lacked the RibA function. Likewise, *ribN* encodes a functional riboflavin importer. Transcriptomics analysis indicated that external riboflavin affected the expression of a relatively small number of genes, including a few involved in iron metabolism. *ribB* was downregulated in response to external riboflavin, suggesting negative feedback. Deletion of *ribA*, *ribB* and *ribE1* showed that these genes are required for *A. salmonicida* riboflavin biosynthesis and virulence in Atlantic lumpfish (*Cyclopterus lumpus*). *A. salmonicida* riboflavin auxotrophic attenuated mutants conferred low protection to lumpfish against virulent *A. salmonicida*. Overall, *A. salmonicida* has multiple riboflavin endowment forms, and duplicated riboflavin provision genes are critical for *A. salmonicida* infection.

## Introduction

Riboflavin or vitamin B_2_ is an essential micronutrient for all forms of life. Riboflavin derivatives, mainly flavin mononucleotide (FMN) and flavin adenine dinucleotide (FAD) (collectively known as flavins), are canonical cofactors for intracellular flavoprotein-mediated reduction/oxidation (RedOx) reactions and play a crucial role in oxidative metabolism [1] [2]. Flavins may also be secreted to participate in extracellular RedOx processes related to bacterial physiology, such as iron reduction, electron transfer for extracellular respiration, the establishment of symbiotic interactions, and quorum-sensing signalling [3–6]. In pathogenic bacteria, riboflavin biosynthesis may also be crucial for virulence during infection [7].

Essential micronutrients are required for successful pathogen infection. Hosts may employ nutritional immunity to limit the availability of micronutrients, such as riboflavin, from systemic circulation and tissues [8,9]. For instance, approximately 60% of the riboflavin in the human plasma is withdrawn as a result of the acute phase response of the host to combat infection [10]. In return, pathogens could either synthesize this vitamin *de novo* through the riboflavin biosynthetic pathway (RBP) or scavenge it from host tissues using flavin transport systems to ensure their proliferation and survival [11]. Energy-wise, riboflavin biosynthesis is more expensive than the uptake [12]. For instance, 25 molecules of ATP are required to synthesize 1 mole of riboflavin, but depending on the transport system, only two or even fewer molecules of ATP are required for uptake [4,13,14]. The RBP produces riboflavin from precursors guanosine-5-triphosphate (GTP) and ribulose-5-phosphate using the activities of five enzymes, GTP cyclohydrolase II (RibA according to the Gram-negative bacteria nomenclature), 3,4-dihydroxy-2-butanone-4-phosphate (3,4-DHBP) synthase (RibB), a bifunctional pyrimidine deaminase/reductase (RibD), riboflavin synthase (RibE) and 6,7-dimethyl-8-ribityllumazine (lumazine) synthase (RibH) [2,11,15–17]. Several bacterial riboflavin uptake systems have been described, and in some species, they coexist with the RBP [11,17,18]. Among them, the RibN transporter is present in Gram-negative proteobacteria such as *Vibrio cholerae* and *Rhizobium leguminosarum* [11,18].

Riboflavin provision pathways in bacteria appear to respond to species-specific metabolic needs of riboflavin [5,7]. When environmental riboflavin is present, riboflavin transporters may substitute for the RBP in riboflavin prototrophs [19–21]. Nonetheless, each riboflavin provision component may have specific, non-redundant functions. For example, in pathogens like *Listeria monocytogenes*, the transporter has been associated with the uptake of specific flavin species during host colonization [22]. So far, little is known about how intraspecies riboflavin supply pathways are coordinated to meet the flavin requirements in bacteria.

Significant differences exist amongst bacteria in the transcriptional organization of the RBP genes. Some species cluster all the RBP genes into a single operon, whereas other species disperse the RBP genes along the chromosome in various transcriptional units [11]. The expression of RBP and transporter genes may be regulated by the FMN riboswitch, a genetic element found upstream of several *rib* operons and monocistronic *rib* genes [18,23–27]. FMN binds to the aptamer portion of the FMN riboswitch, inhibiting the transcription and or translation of the downstream genes [2,23].

In bacteria, some RBP enzymes may have duplicate or multiple gene copies. These gene duplications provide bacteria more flexibility in how they genetically manage their riboflavin supply [11]. In general, gene duplication events that are maintained in a population have benefits that surpass the fitness cost of carrying the duplication [28]. Intra-genome conserved multiple gene copies may confer adaptive advantages to the bacteria, such as improvements in their ability to adjust to changing environmental conditions (i.e. *ex vivo* and *in vivo*) [29]. Therefore, the extra copies of the RBP genes may have specific functions and provide adaptive benefits [11]. For instance, *Brucella abortus* has a second *ribH* gene outside of the main RBP operon that is directly linked to intracellular survival and host colonization [7]. Overall, the variation in the assortment of copies of RBP genes and riboflavin importers may impact bacterial virulence and physiology.

The bacterial riboflavin provision pathways have been studied in some important human and animal pathogens, and the genes that encode for their RBP and riboflavin transporters have been identified [11,30–32]. However, this knowledge is lacking in marine pathogens of fish like *A. salmonicida*, which causes significant economic losses in finfish aquaculture. *A. salmonicida* is the aetiological agent of furunculosis in various fish species [33,34], such as lumpfish (*Cyclopterus lumpus*), which is a cleaner fish employed to biologically control the sea lice (*Lepeophtheirus salmonis*) infestations in Atlantic salmon (*Salmo salar*) sea cages [35–38]. As a psychrotropic waterborne pathogen, *A. salmonicida* infects marine and freshwater fish [33,34]. The economic importance, suitability for genetic manipulation, and relatively reproducible *in vivo* infection make *A. salmonicida* a good model organism to study psychrotropic marine pathogenesis [34,39–42]. Vaccines and antimicrobials have often been employed to prevent or control *A. salmonicida* disease outbreaks in aquaculture [43,44]. However, furunculosis still persists in some cultured fish species due to low vaccine or antimicrobial efficacy [45,46]. A better understanding of *A. salmonicida* physiology and virulence mechanisms is needed to design more adequate treatments or preferably, efficient vaccines to avoid its pernicious effects in aquaculture and spillbacks to wild fish. Given the importance and divergent effects of riboflavin in bacteria, in this study, we aimed to identify the riboflavin provision systems in *A. salmonicida* and their role in different physiological traits and virulence.

In the present study, we determined the presence and characterized the transcriptional organization of riboflavin supply pathways in *A. salmonicida*, using genomic information and experimental characterization. A composite RBP featuring redundant functions and riboflavin uptake are present in *A. salmonicida*. The role of the different riboflavin provision components in virulence, the general regulatory effects of external riboflavin, and the possible use of flavin-impaired mutants as attenuated vaccines were also explored. Overall, this study characterizes the riboflavin provision pathways of *A. salmonicida* and starts elucidating their contribution to pathogenicity in a cold water marine teleost.

## Materials and methods

### Bacterial strains, plasmids, media and reagents

*A. salmonicida* wild-type J223 strain isolated from Atlantic salmon was used in this study [40] (Table 1). This isolate served as the source for all genetically defined *A. salmonicida* mutants constructed in this study (Table 1). Table 1 includes information about bacterial strains and plasmids. Media for bacteriology were from Difco (Franklin Lakes, NJ, USA). Sigma-Aldrich (St Louis, MO, USA) supplied the antibiotics, riboflavin, and reagents. Trypticase Soy Broth (TSB), M9 minimal media adapted for *A. salmonicida* growth (33 mM Na_2_HPO_4_, 22 mM KH_2_PO_4_, 20 mM NH_4_CI, 10 mM NaCl, 1 mM MgSO_4_, 0.1 mM CaCl_2_, 10 mM glucose, 0.25 mM L-arginine and 0.25 mM L-methionine) [50], and Luria Bertani (LB) broth (tryptone 10 g; yeast extract 5 g; NaCl 10 g; dextrose 1 g; double distilled water, 1 L) [51] were routinely used. The media were supplemented as necessary with riboflavin (2 or 500 μM) [21], 1.5% agar, 10 % sucrose, Congo red (50 μg/mL), chloramphenicol (Cm; 25 μg/mL), kanamycin (Km; 50 μg/mL), gentamicin (Gm; 10 μg/mL), ampicillin (Amp; 100 μg/mL) or diaminopimelic acid (DAP; 50 μg/mL). *A. salmonicida* J223 and mutant strains were routinely cultured in TSB or modified M9 minimal media at 15 ºC with aeration (180 rpm). *Escherichia coli* wild-type and mutant strains were cultured in LB at 37 °C with aeration (180 rpm). Spectrophotometry and/or agar plate counting were used to track bacterial growth. Primers used in this study (Table S1-S3) were synthesized by Integrated DNA Technologies (IDT, San Diego, CA, USA). Restriction endonucleases were from New England Biolabs (Whitby, ON, Canada). All PCR assays were conducted using GoTaq Green Master Mix from Promega (Madison, WI, USA). T4 ligase and T4 DNA polymerase were from Promega (Madison, WI, USA). Plasmid DNA was isolated, and gel DNA fragments and PCR products were purified using Qiagen products (Germantown, MD, USA).

**Table 1.** List of strains and plasmids used in this study.

Strains/plasmids	Characteristics	Source
*Aeromonas salmonicida*		
J223	Wild type	[40]
J412	Δ*ribA*; J223 derivate	This study
J413	Δ*ribB*; J223 derivate	This study
J414	Δ*ribBA*; J223 derivate	This study
J415	Δ*ribE1*; J223 derivate	This study
J416	Δ*ribE2*; J223 derivate	This study
J417	Δ*ribN*; J223 derivate	This study
J418	Δ*ribA*-Δ*ribE1*; J223 derivate	This study
*Escherichia coli*		
χ*7213*	*thr-1 leu*B6 *fhu*A21 *lac*Y1 *glnV44 recA1* Δ*asdA4* Δ(*zhf*-2:Tn*10*) *thi-1* RP4–2-Tc:Mu [λ*pir*]; Km^r^ Tet^s^ Amp^s^ DAP–	[47]
χ*7232*	*endA*1 *hsdR*17 (r_k_-, m_k_+) *supE44 thi-1 recA*1 *gyrA relA*1 Δ(*lac*ZYA-*argF*) U169 λ*pir deoR* (φ80dlacΔ(*lacZ*)M15); Nal^r^ UV^s^ Thi – Lac–	[48]
BW25113 ∆*ribB*	Wild-type *Escherichia coli* BW25113 ∆*ribB*::kan	[49] [18]
∆*ribA*	*E. coli* DH5α ∆*ribA*::cat	This study
Plasmids		
pR112	5173 bp, Suicide vector Cm, *sacB*, *oriV*, *oriT*	[48]
pMEG-375	8142 bp, Suicide vector, Cm, Amp, *lacZ*, R6K *ori*, *mob incP, sacR sacB*	[48]
TOPO	3.9 kb, pUC ori, Km^r^, Amp^r^	Invitrogen
pEZ323	pR112 derivate; Δ*ribA*	This study
pEZ324	pR112 derivate; Δ*ribB*	This study
pEZ325	pR112 derivate; Δ*ribBA*	This study
pEZ326	pMEG-375 derivate; Δ*ribE1*	This study
pEZ327	pMEG-375 derivate; Δ*ribE2*	This study
pEZ328	pR112 derivate; Δ*ribN*	This study
pEZ329	pTOPO-*ribBA*-Asal; Plasmid bearing *ribBA* of *A. salmonicida*, P_lac_-*ribBA*, amp^r^ Km^r^	This study
pEZ330	pTOPO-*ribB*-Asal; Plasmid bearing *ribB* of *A. salmonicida*, P_lac_-*ribB*, amp^r^ Km^r^	This study
pEZ331	pTOPO-*ribN*-Asal; Plasmid bearing *ribN* of *A. salmonicida*, P_lac_-*ribN*, amp^r^ Km^r^	This study
pKD46	Plasmid expressing the λ-red recombinase system, amp^r^	[49]
pKD3	Template plasmid bearing kanamycin resistance cassette, Km^r^	[49]
pG-*ribA*-Eco	Plasmid bearing *ribA* of *E. coli*	This study
pG-*ribB*-Vch	Plasmid bearing *ribB* of *Vibrio cholerae*	[21]

### *In-silico* characterization of riboflavin supply pathways and genes in *A. salmonicida*

*A. salmonicida* A449 and J223 genomes from the National Center for Biotechnology Information (NCBI) [52] and Kyoto Encyclopedia of Genes and Genomes (KEGG) [53] public databases were utilized for the search of RBP and transport genes and their organization within the genome. The RibEx tool was utilized to identify putative FMN riboswitches [54]. Amino acid sequences of the RBP proteins and their duplicated or multiplicated copies were obtained in FASTA format from NCBI. Protein sequence alignments were performed in Jalview (Version 2.11.2.5) platform (www.jalview.org) [55] using web service function for Clustal Omega Multiple Sequence Alignment Program with default paraments, and sequence similarity values (i.e. percent identity and percent similarity) were obtained. Aligned sequences were rendered using the web-based interface of Easy Sequencing in PostScript (ESPript) [56]. The tridimensional (3D) protein structures of the RBP enzymes and their additional copies were modelled in HHpred [57] and visualized in Visual Molecular Dynamics (VMD; Version 1.9.1) [58]. Structure predictions for the duplicated enzymes were conducted using trRosetta modelling (https://yanglab.nankai.edu.cn/trRosetta/) [59]. 3D protein structures were compared and overlapped in VMD using the Structural Alignment of Multiple Proteins (STAMP) tool, and the structural homology values (Q_H_) and root mean square deviation (RMSD) were generated.

### Experimental characterization of riboflavin supply pathways in *A. salmonicida*

#### Bacterial growth in minimal media

Three millilitres of TSB were inoculated with a single colony of *A. salmonicida* J223 and incubated at 15 ºC overnight in a roller drum (TC-7, New Brunswick Scientific Co, San Diego, NJ, USA). Then, 100 µL of the *A. salmonicida* overnight culture were transferred into 3 mL of modified M9 minimal media and allowed to grow for 2–3 days at 15 ºC in a roller drum. Next, 30 µL of this minimal media culture were transferred into 3 mL of fresh minimal media and allowed to grow for another 2–3 days at 15 ºC in a roller drum. Finally, the minimal media culture was centrifuged at 10,000 rpm for 2 minutes at 4 ºC, the supernatant was discarded, the pellet was washed 3 times with minimal medium, and resuspended in 1 mL fresh minimal media. Three-hundred microlitres of the resuspended cells were inoculated into three 50 mL individual flasks having 30 mL of fresh minimal media. Cultures were incubated at 15 ºC with aeration (180 rpm) in an orbital shaker (MaxQ 4000, Thermo Fisher Scientific, MA, USA) until an optical density (OD_600 nm_) of 0.7 (~1×10^8^ Colony Forming Units per mL (CFU/mL)). Next, these triplicate cultures were subjected to RNA extraction using established protocols [60].

#### Total RNA extraction, Reverse Transcription and Polymerase Chain Reaction (RT-PCR)

Once the *A. salmonicida* cultures (*n* = 3) reached the desired OD_600 nm_, cells were extracted by centrifugation (6000 rpm for 10 min) at 4 ºC and twice washed with phosphate-buffered saline (PBS, pH 7.0; 136 mM NaCl, 2.7 mM KCl, 10.1 mM Na_2_HPO_4_, 1.5 mM KH_2_PO_4_) [61]. The cell pellets were used for RNA extraction. TRIzol reagent (Invitrogen) was used to extract total RNA, and the RNeasy MinElute Cleanup Kit was used to purify it (Qiagen, Mississauga, ON, Canada) using the manufacturer’s guidelines. RNA extracts were digested with TURBO DNA-free™ Kit (Invitrogen, Carlsbad, CA, USA). Purified RNA samples were measured using a Genova Nano microvolume spectrophotometer (Jenway, UK), and 1% agarose gel electrophoresis was used to verify the samples’ integrity [61].

To experimentally depict the transcriptional orchestration of the *A. salmonicida* riboflavin supply genes, an RT-PCR was performed, as explained before by Cisternas et al. (2017). cDNA was synthesized using SuperScript Vilo IV Master Mix with reverse transcriptase (Invitrogen) as directed by the manufacturers’ instructions with 1 µg RNA per reaction. PCR assays were carried out on these cDNAs using the primers (Table S1) that amplify the putative gene junctions. For each sample (*n* = 3), a control reaction without reverse transcriptase (negative control) was added. Positive controls included PCR reactions on the *A. salmonicida* J223 genomic DNA with respective primers. Following the amplifications, the putative gene junctions tested were visualized in 1% agarose gel electrophoresis [61]. Positive PCR amplifications in this approach imply the joint of coding sequences in the same messenger RNA (mRNA), therefore, the genes are adjacent to each other and form an operon [21].

### A. salmonicida gene functionality assays

#### Construction of complementation plasmids with *A. salmonicida ribB, ribBA*, *ribN*

The *A. salmonicida* genes *ribB*, *ribBA*, and *ribN* were independently cloned into high copy number plasmid (pCR™2.1-TOPO™) (Table 1) under P_lac_ control at the *Adh*I restriction site. Table S3 provides the list of primers utilized to amplify the corresponding genes under P_lac_ control. The resultant plasmids were used to complement the *E. coli* riboflavin auxotrophic mutant strains (Table 1).

#### Construction of *E. coli ΔribA* mutant and complementation plasmid

The *E. coli ∆ribA* null mutant was constructed according to the mutagenesis by homologous recombination with PCR fragments protocol described before [49]. *E. coli* BW25113 bearing pKD46, previously grown at 30°C with arabinose was electroporated with a PCR product obtained with primers *E. coli-ribA*-H1P1 and *E. coli-ribA*-H2P2 (Table S3) and the pKD3 plasmid as template DNA. Candidate recombinant mutants were selected in LB plates with Cm and incubated overnight at 42 °C. The candidates obtained were screened by PCR for the replacement of *ribA* by the Km resistance cassette using primers flanking the recombination site *E. coli*-RibA-Fw and *E. coli*-RibA-Rv (Table S3).

The plasmid pGEco*ribA* to complement *E. coli ribA* mutants was constructed by ligating a PCR product obtained with the set of primers *E. coli*-RibA-Fw/*E. coli*-RibA-Rv and wild-type *E. coli* genomic DNA in pGEM T Easy (Promega) in accordance with the manufacturer´s protocol.

#### Functional complementation analysis in E. coli heterologous model

After transferring the complementing plasmid vectors into *E. coli* riboflavin auxotrophic mutants of Δ*ribA* and Δ*ribB* (Table 1), the phenotypic rescue of the *E. coli* mutants was evaluated either in LB or M9 minimal media agar plates in the presence and absence of riboflavin, to assess the functional complementation of riboflavin biosynthesis. Briefly, overnight cultures of *E. coli* wild-type and its derivative mutants and complemented strains grown in LB with 500 µM riboflavin were washed twice with plain LB or M9 and resuspended in fresh media without added riboflavin. These cultures were then serially diluted and 5 µL were spotted into LB or minimal media plates supplemented with 500 µM riboflavin, 2 µM riboflavin or without riboflavin. Plates were incubated at 37 °C overnight to observe growth.

### *A. salmonicida* transcriptomics and qPCR analyses

#### Bacterial growth in minimal media with and without riboflavin, RNA extraction and cDNA synthesis

Fifty millilitres of *A. salmonicida* wild-type J223 cultures grown with (2 µM) and without riboflavin at 15°C with shaking (180 rpm) to an OD_600 nm_ of 0.7 were subjected to TRIzol lysis, RNA extraction (*n* = 6), column-purification and DNase treatment, as previously described [60]. These RNA samples were used for RNA sequencing and RT-qPCR (Figure S1). High-Capacity cDNA Reverse Transcription Kit (Thermofisher, Foster City, CA, USA) was used to obtain first-strand cDNA templates for qPCR from 1 µg purified RNA in 20 µL reactions, as instructed by the manufacturer.

#### Library preparation and RNA sequencing

For each experimental condition (Control (*n* = 3) and riboflavin-supplemented (*n* = 3) groups), there were 3 biological replicates (Total *n* = 6). Genome Quebec, Canada carried out the commercial library construction and RNA sequencing. Briefly, RNA quality was assessed using a Bioanalyzer 2100 (Agilent). rRNA was depleted using NEBNext® rRNA Depletion Kit (Bacteria). cDNA libraries were constructed using the adapters and primers of NEBNext® Multiplex Oligos for Illumina®. Sequencing was performed on a NovaSeq 6000 (Illumina) platform with a 100 bp paired-end protocol. Raw sequencing data has been submitted in the NCBI Bio Project database under the accession number PRJNA909183.

#### RNA-seq data analyses

Data from RNA-seq were analysed in CLC Genomics Workbench v22.0 (CLCGWB; Qiagen, Hilden, Germany) using comparable settings as those previously disclosed [62,63]. Low quality reads were removed, and clean paired reads were generated. The trim read tool in CLCGWB was used to trim the adapters with the default criteria. Quality control visualization of the reads was performed using FastQC (https://www.bioinformatics.babraham.ac.uk/projects/fastqc/) and multiQC [64] before and after trimming. The RNA-seq analysis program was used by CLCGWB to map good-quality trimmed reads to the A. salmonicida genome (Accession: PRJNA310296). The gene abundance of mapped reads was quantified and normalized using RSEM and eXpress approaches [65,66]. The transcript per million reads (TPM) values were then determined using the counts ascribed to each transcript [67]. A global correlation analysis, using the Pearson method to quantify the correlation, was performed on the Log2 TPM values (x + 1) for individual gene under the presence and absence of riboflavin conditions. Abundance data were consequently exposed to differential expression analysis in CLCGWB with negative binomial general linear model-based (GLM) normalization [68]. Biologically relevant differentially expressed genes (DEGs) were identified using the standard cut-off values of log2 fold-change (FC) ≥ |1| and false discovery rate (FDR) p ≤ 0.05. The expression folds (in terms of TPM values) of significant DEGs from control and riboflavin-supplemented groups were compared and visualized in bar plots using GraphPad Prism 7.0 (GraphPad Software, La Jolla, California, USA). Operon mapper was used to predict whether the contiguous DEGs formed operons (http://biocomputo.ibt.unam.mx/operon_mapper/) [69]

#### Gradient PCR

Primers used for each gene (i.e. *ribA*, *ribBA*, *ribB*, *ribD*, *ribE1*, *ribE2*, *ribH*, and *ribN*) for the real-time quantitative PCR (RT-qPCR) analysis are stated in Table S2. Gradient PCR was carried out to determine the ideal annealing or melting temperature (T_m_) for each primer set, as described by Conners et al. (2019). Finally, specific amplicons for each primer set were visualized in 1% agarose gel. Gradient PCR demonstrated that all RT-qPCR primers (Table S2) amplified a single amplicon at an ideal T_m_ of 55–60 °C (Figure S2A).

#### RT-qPCR

To examine how the extracellular riboflavin affects the expression of the transcriptional units that encode for riboflavin supply pathways, the RT-qPCR amplifications were carried out, as previously mentioned [60]. Primer pairs for riboflavin supply genes were designed, and the standard curve approach was used to determine the primer efficiency and confirm the primer specificity (Table S2). A cDNA pool made from control (*n*=3) and riboflavin-supplemented (2 µM; *n*=3) *A. salmonicida* cultures in minimal media was used to analyze the primer efficiencies. Pooled cDNA was serially diluted with a 5-point 1:3 dilution series beginning at 20 ng/L. Amplification efficiencies were estimated according to Pfaffl, 2001 [70]. Information on RT-qPCR primers are listed in Table S2. Each pair of primers' melt curves had a single peak, proving that they did not produce dimers and that they were specific for a single amplicon (Figure S2B).

To select two endogenous control genes for RT-qPCR, 5 genes (i.e., *hfq*, *era*, *rpoB*, *recA*, and *fabD*) that had been reported in *A. salmonicida* to normalize transcriptional expression data were analyzed [60]. Raw threshold cycle (C_T_) values of all 6 samples were determined in triplicates for each of these genes using cDNA equivalent to the input total RNA of 20 ng. The observed Ct value ranges were consistent and acceptable (i.e., between 20-30) independent of the condition being tested (Figure S2C). The geNorm tool in the Ref-Finder open-access portal was used to examine the stability of these genes’ expression [71]. *era* (geNorm M = 0.223) and *hfq* (geNorm M = 0.543) were chosen as the two endogenous controls based on their constitutive expression. 

All RT-qPCR reactions were performed using cDNA (5 ng/μL) and Power SYBR™ Green Master Mix (Applied Biosystems, Carlsbad, CA, USA) in QuantStudio 3 (Applied Biosystems) with the experimental qPCR parameters described in Conners et al. (2019). Each experimental condition was evaluated with biological (*n* = 3) and technical (*n* = 3) triplicates. Relative gene expression levels were estimated using the comparative 2^−ΔΔCt^ method [70,71].

To further evaluate the correlation between gene expression levels from RNA-seq and RT-qPCR data, a simple linear regression analysis was performed between the normalized counts (TPM) of RNA-seq data (Log_2_ TPM on the X axis) and the Ct values from RT-qPCR (Log_2_ Ct on the Y axis), and the Pearson correlation coefficients (r^2^; *p* < 0.05) were calculated.

### *A. salmonicida* mutants’ construction and characterization

In-frame deletion of *ribA*, *ribB*, *ribBA*, *ribE1*, *ribE2*, *ribN* genes in *A. salmonicida* was accomplished using recombinant suicide vectors (Table 1) bearing the joined flanking regions, as previously reported [40,72]. A deletion with the ATG start codon but without the TAG or TAA stop codon is contained in the defined deletion mutations. Methods for PCR, DNA isolation, DNA cloning, restriction enzyme digestion, and plasmid construction are standard [61]. Table S3 contains a list of all primers used for the mutant construction. Primer sets F1-R1 and F2-R2 were designed to amplify the up- and down-stream flanking regions, respectively. The flanking regions were amplified from *A. salmonicida* J223. Overlapping PCR was used to ligate the flanking regions. The PCR products containing in-frame deletion fragments of the selected genes were cloned into either pR112 or pMEG375 (Table 1) that had been digested with *Sph*I and *Xba*I. To construct *A. salmonicida* single deletion mutants (Δ*ribA*, Δ*ribB*, Δ*ribBA*, Δ*ribE1*, Δ*ribE2*, Δ*ribN*), the suicide plasmid was transferred from *E. coli* χ7213 to *A. salmonicida* J223 by conjugation. To construct *A. salmonicida* double mutant (Δ*ribA*-Δ*ribE1*), the suicide plasmid carrying in-frame deletion fragment of *ribA* gene (i.e. pEZ323; Table 1) was conjugationally transferred from *E. coli* χ7213 to *A. salmonicida* J415 (i.e. Δ*ribE1*). The transconjugants, in which the single-crossover plasmid insertions homologously recombined into the chromosome, were selected on TSA plates having Cm. The second recombination within homologous regions (i.e. allelic exchange) that results in the loss of suicide vector was selected by employing the “*sacB*-based sucrose sensitivity counter-selection system” adapted to *A. salmonicida* [40,73,74]. The colonies were chosen for Cm^r^ and screened by PCR with the use of primers F1 and R2.

Growth curve and biochemical profile assays were performed to characterize the phenotypic and biochemical differences in bacterial physiology between *A. salmonicida* J223 wild-type and mutant strains. Growth of *A. salmonicida* strains was evaluated in minimal media in the presence and absence of riboflavin at 15 ºC in triplicates. Briefly, *A. salmonicida* strains were grown in 3 mL of M9 minimal media as previously described in section 2.3.1. 300 µL of these cultures were inoculated into 100 mL flasks containing 50 mL of fresh minimal media with and without riboflavin (2 µM) and incubated at 15ºC with shaking (180 rpm) for 15 days. Bacterial growth was monitored spectrophotometrically until the OD_600 nm_ readings were stabilized (OD_600 nm_~1 to 1.5). Biochemical and enzymatic profiles of *A. salmonicida* strains were determined using the API20E, API20NE, and APY-ZYM (BioMerieux, Marcy-l’Etoile, France) as instructed by the manufacturer. Stripes were incubated with *A. salmonicida* strains at 15ºC for 48 h, and the API WEB (BioMerieux) was used to examine the results.

### Evaluation of *A. salmonicida* virulence in lumpfish (*C. lumpus*)

#### Bacterial inocula preparation

The bacterial inocula for infection and challenge were prepared according to the prior instructions with minor modifications [40]. Briefly, *A. salmonicida* J223 and mutant strains were cultured in 3 mL of TSB at 15 ºC in a roller drum overnight. Three-hundred microliters of these cultures were inoculated into 100 mL flasks comprising 30 mL of fresh TSB and incubated at 15 ºC with shaking (180 rpm) up to an OD600nm of 0.7 (~1x108 CFU/mL). By centrifuging at 6000 rpm for 10 min, at 4 ºC, bacterial cells were collected, washed once with PBS, and resuspended in 300 µL of PBS. The bacterial cell suspension was serially diluted in PBS (1:10) to achieve the final infection and challenge doses, at the same time, enumerated by plating on Trypticase Soy Agar (TSA) to determine CFU/mL.

#### Fish holding

This study was conducted using animal protocols that were approved by the Institutional Animal Care Committee and the Biosafety Committee at Memorial University of Newfoundland (MUN) (https://www.mun.ca/research/about/acs/acc/) in accordance with the guidelines set by the Canadian Council on Animal Care (https://ccac.ca/). The protocols #18–01-JS, #18–03-JS, and biohazard licence L-01 were used for the fish experiments. Lumpfish (55.4±5.6; mean±SD) were maintained at the Joe Brown Aquatic Research Building (JBARB), Department of Ocean Sciences (DOS), MUN and transferred to the aquatic level 3 (AQ3) biocontainment unit at the Cold-Ocean Deep-Sea Research Facility (CDRF), DOS, MUN for infection assays.

Fish were kept in ideal conditions before and during the experiment, including 500L circular tanks with flow-through seawater system (7.5L/min) using filtered, UV-treated seawater at 8–10 °C, 95–110% oxygen saturation and ambient photoperiod (12h light: 12h dark) (Figure S3). Biomass density was kept at 25kg per m^3^. Fish were fed daily with commercial aqua-feed (Skretting – Europa 15) at a rate of 0.5% of fish body weight per day.

### Infection and challenge

Lumpfish from JBARB were divided into nine 500L tanks containing 60 fish per tank at CDRF and acclimatized for 2 weeks before infection. The fish were sedated in 40mg/L of tricaine methane-sulphonate (MS-222; Syndel Laboratories, Vancouver, BC, Canada), and intraperitoneally (ip) infected with either 100µL of PBS or 100µL (10^4^ CFU/dose) of wild-type *A. salmonicida* J223, and mutant strains (Figure S3). Fish were observed daily for mortality and clinical signs until 30 days post-infection (dpi). Finally, surviving fish at 30 dpi were ip challenged with 10^3^ CFU/dose (10 LD_50_ (lethal dose 50%)) of *A. salmonicida* J223 wild-type.

### Colonization of *A. salmonicida* wild-type and mutants in lumpfish tissues

A MS222 overdose (400mg/L) was used to euthanize five lumpfish (*n*=5) that were randomly selected at 3, 7, and 10 dpi. Samples of the spleen, liver, head kidney, and brain were aseptically removed and individually placed into sterile homogenizer bags (Nasco whirl-pak®, USA). Next, tissue samples were weighed, and homogenized in PBS to achieve a final volume of 1mL (weight: volume; 0.1g of tissue per 1mL of PBS). These tissue suspensions were then serially diluted (1:10) and counted on TSA-Congo red (TSA-CR) plates. Likewise, 1mL of blood was drawn, serially diluted, and plate counted onto TSA-CR. Wild-type *A. salmonicida* J223 or mutants CFU per g of tissue or per mL of blood were counted on the plates after 4–5 days of incubation at 15 °C.

### Statistical analyses

The Prism program version 7.0 was used to conduct statistical analyses and visualize the data. A *p*-value≤0.05 was considered statistically significant. Non-parametric one-way ANOVA Kruskal-Wallis test followed by Dunn’s multiple comparison post-hoc test was used to identify significant differences in the gene expression between groups (i.e. control and riboflavin-supplemented *A. salmonicida*). Kaplan-Meier estimator and Log-rank test were employed to obtain survival fractions following infection and to compare survival curve trends, respectively. A one-way ANOVA with a non-parametric Kruskal-Wallis test was used to compare the tissue colonization, and Dunn’s multiple comparison post-hoc analysis was utilized to determine the significant differences in colonization between *A. salmonicida* J223 wild-type and mutant strains.

## Results

### *A. salmonicida* encodes a full RBP with additional ribB and *ribE* copies and a *RibN* riboflavin transporter

**Figure 1.**
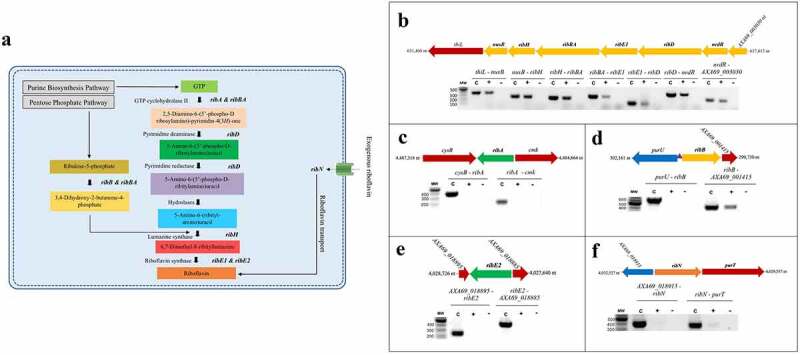
*In-silico* and experimental characterization of riboflavin supply pathways in *A. salmonicida*. A. Schematic illustration of riboflavin provision pathways: Riboflavin Biosynthetic Pathway (RBP) and RibN family transporter. (B-F). Evaluation of the transcriptional organization of *rib* genes in *A. salmonicida*. PCR reactions were performed on *A. salmonicida* cDNA using primers that amplify the specified gene junctions of *rib* genes and their adjacent genes at the loci encoding *ribD*, *ribE1*, *ribBA, ribH* (B), *ribA* (C), *ribB* (D), *ribE2* (E) and *ribN* (F). Each reaction was carried out 3 times separately with the same results. The template cDNA from RT-PCR with reverse transcriptase is indicated by **+**, while the template cDNA from RT-PCR without reverse transcriptase is indicated by - (negative control). C implies PCR on chromosomal DNA as the template (positive control) and M stands for molecular weight marker in base pairs. The purple triangle between *purU* and *ribB* indicates the presence of FMN riboswitch. .

The enzymatic steps of the RBP and the associated catalytic enzymes are depicted in Figure 1a. The biochemical pathway of riboflavin synthesis utilizes one molecule of Guanosine-5’-triphosphate (GTP) resulting from the purine biosynthesis pathway and two molecules of ribulose-5-phosphate from the pentose phosphate pathway to yield one molecule of riboflavin after a series of enzyme-catalysed reactions (Figure 1a). To identify the riboflavin provision genes of *A. salmonicida* J223, we searched its genome in the NCBI and KEGG databases for RBP genes and riboflavin transporters and their functions, which are listed in Table 2. The results of this search indicated that *A. salmonicida* conserves a cluster of contiguous *ribD*, *ribE*, *ribBA*, *ribE1*, and *ribH* genes localized between *nusB* and *nrdR* (Figure 1b). Theoretically, this cluster would encode all enzymes required for riboflavin biosynthesis, with the *ribBA* gene product annotated as a fusion of the RibB and RibA proteins. In addition to this main cluster, a copy of *ribE*, denominated here as *ribE2*, and copies of independent *ribA*, *ribB* and a *ribN* gene encoding a putative riboflavin transporter were identified in different regions of the chromosome (Figures 1c–f). Riboswitch prediction in the putative regulatory regions of the identified genes using RibEx [54] indicated that the upstream region of *ribB* contains a conserved FMN riboswitch (Figure 1d).

**Table 2.** Riboflavin supply genes in *A. salmonicida*.

Gene	Function	Location	Size (nt)	Locus tag
*ribA*	GTP cyclohydrolase II	Gene; 4485605.4486198	594	AXA69_020950
*ribB*	3,4-dihydroxy-2-butanone-4-phosphate synthase	Gene; complement 300,198.300851	654	AXA69_001420
*ribBA*	Bifunctional 3,4-dihydroxy-2-butanone-4-phosphate synthase/GTP cyclohydrolase II	Operon; complement 633,551.634,660	1110	AXA69_003010
*ribD*	Bifunctional diamino hydroxy phosphoribosyl amino-pyrimidine deaminase/5-amino-6-(5 phosphoribosyl amino)uracil reductase ribD	Operon; complement 635,513.636,622	1110	AXA69_003020
*ribE1*	Riboflavin synthase	Operon; complement 634,804.635,457	654	AXA69_003015
*ribE2*	Riboflavin synthase	Gene; 4027977.4028594	618	AXA69_018890
*ribH*	6,7-dimethyl-8-ribityllumazine synthase	Operon; complement 632,949.633,419	471	AXA69_003005
*ribN*	DMT family transporter	Gene; complement 4,030,823.4,031,713	891	AXA69_018910
*nusB*	Transcription antitermination factor nusB	Operon; complement 632,521.632,934	414	AXA69_003000
*nrdR*	Transcriptional regulator nrdR	Operon; complement 636,697.637,146	450	AXA69_003025

To experimentally characterize the transcriptional organization of the *A. salmonicida* riboflavin supply genes (Table 2), PCR analyses were conducted on cDNA obtained from RNA of *A. salmonicida* J223 cultured in minimal media with primers designed to amplify the gene junctions (Table S1). Positive amplifications in the RT-PCR imply the joint of coding sequences in the same mRNA. Results revealed that the gene cluster composed of adjoining *nusB*, *ribH*, *ribBA*, *ribE1*, *ribD*, and *nrdR* was part of an operon (Figure 1b). Moreover, this operon also includes the genes *thiL* and A×A69_003030 (*NIPSNAP* [4-nitrophenyl phosphatase and non-neuronal SNAP25] family protein), which have putative roles in thiamine biosynthesis and vesicular transport, respectively, localized contiguous to *nusB* and *nrdR*, respectively (Figure 1b). This analysis showed that *ribA* comprises a monocistronic unit (Figure 1C) while *ribB* forms an operon with the downstream open reading frame (ORF) A×A69_001415 gene encoding a putative Lpp/OprI family alanine-zipper lipoprotein (Figure 1d). The second copy of the riboflavin synthase-encoding gene *ribE2*, located outside the main RBP operon, is a monocistronic unit (Figure 1e). Finally, this analysis showed that the riboflavin transporter *ribN* gene forms an operon with *purT*, which encodes a putative formate-dependent phosphoribosyl glycinamide formyl transferase (Figure 1f).

**Figure 2.**
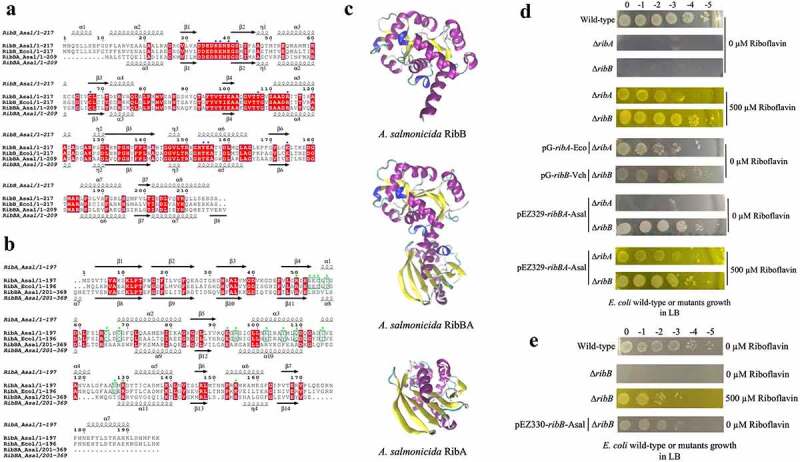
Sequence alignment, three-dimensional (3D) protein structures and functionality of *ribB*, *ribA*, and *ribBA* (or *ribBX*) genes. Amino acid sequences of experimentally resolved RibA and RibB proteins from *E. coli* are used. RibA and RibB active site residues are identified using reported literature. Conserved amino acid residues are highlighted in red. a. Multiple sequence alignment among RibB of *E. coli* (*Ecol*), RibB and amino-terminal region of the RibBA fusion (amino acids 1 to 207) of *A. salmonicida* (*Asal*). The secondary structures at the top and bottom of the alignment correspond to the *A. salmonicida* RibB and RibBA, respectively (spirals represent α-helix; arrows represent β-sheet). The blue circles at the top of the aligned sequence indicated the key catalytic active site residues (*n* = 11) that have been described in *E. coli* RibB, which are also present in *A. salmonicida* RibB and RibBA. b. Multiple sequence alignment among RibA of *E. coli* (*Ecol*), RibA, and carboxyl-terminal region of the RibBA fusion (amino acids 204 to 369) of *A. salmonicida* (*Asal*). The secondary structures at the top and bottom of the alignment correspond to the *A. salmonicida* RibA and RibBA, respectively. The green circles at the top of the aligned sequence indicated the key catalytic active site residues (*n* = 16) that have been described in *E. coli* RibA. Out of these 16 active sites, 4 are conserved in both RibA and RibBA of *A. salmonicida*, while the remaining 12 are absent from *A. salmonicida* RibBA but conserved in RibA (highlighted with green squares). c. 3D protein structures of *A. salmonicida* RibB, RibBA, and RibA. 3D structures are predicted using the trRosetta protein structure prediction service and visualized in VMD. d. Complementation of *E. coli ribA* and *ribB* mutants with *ribBA* fusion gene of *A. salmonicida* in LB with no (0 µm riboflavin) and 500 µm riboflavin. e. Complementation of *E. coli ribB* mutant with *ribB* of *A. salmonicida* in LB with no (0 µm riboflavin) and 500 µm riboflavin.

According to the first insights from *in silico* and experimental analysis, *A. salmonicida* J223 possesses a full riboflavin biosynthetic pathway with possible duplications in RibA (GTP-cyclohydrolase II), RibB (3,4-DHBP synthase) and RibE (riboflavin synthase) activities, together with a RibN riboflavin transporter. To characterize the functionality of the possible duplicated genes, the protein sequences of putative orthologs were analysed. First, the sequences of the RibBA fusion and the standalone RibB of *A. salmonicida* were aligned to the fully characterized *E. coli* RibB [75]. This alignment analysis indicated that *E. coli* RibB shares 51.69% identity with the amino half of the *A. salmonicida* RibBA fusion (amino acids 1 to 207). Both *A. salmonicida* RibBA and RibB conserve the critical residues for the 3,4-DHBP synthase activity characterized in RibB from *E. coli* (Figure 2a). Next, *A. salmonicida* RibBA and RibA were aligned together with RibA from *E. coli*. This alignment showed that *E. coli* RibA shares 31.40 % identity with the carboxyl-terminal domain of *A. salmonicida* RibBA (amino acids 204 to 369). Nonetheless, while the monofunctional *A. salmonicida* RibA protein conserves the 16 critical residues for GTP cyclohydrolase II activity described in *E. coli* RibA [76], the corresponding domain of *A. salmonicida* RibBA conserves only four of them (Figure 2b). Thus, *in-silico* sequence analysis suggests that the product of *A. salmonicida ribBA* (Figure 2c) in the main riboflavin biosynthetic operon possesses RibB activity but lacks RibA activity. Subsequently, we evaluated the ability of *A. salmonicida ribBA* fusion to complement *E. coli ribA* and *ribB* null mutants. Both *E. coli* ∆*ribA* and ∆*ribB* (Table 1) are riboflavin auxotroph strains that require a high riboflavin concentration (500 µM) to grow in LB (Figure 2d). Control plasmids expressing *E. coli ribA* or *V. cholerae ribB* [77] complemented the growth of their respective *E. coli* riboflavin auxotroph mutant strains in LB without added riboflavin (Figure 2d). In line with the insights obtained from the alignments, *A. salmonicida* RibBA fusion was able to complement the growth of the *E. coli* ∆*ribB* but not that of the ∆*ribA* (Figure 2d). Growth of *E. coli ribA* harbouring the plasmid encoding *A. salmonicida* RibBA was only achieved in high riboflavin concentration (Figure 2d). These results indicated that the fusion annotated as RibBA in *A. salmonicida* does not conserve the GTP-cyclohydrolase II activity. Thus, this gene likely belongs to a family of previously identified genes encoded in RBP operons in different bacteria that encode for a fusion of a functional RibB and a domain of unknown function denominated RibBX [78,79]. Hence this gene was denominated *ribBX* hereafter.

A plasmid expressing the independent *E. coli ribA* gene complemented the growth of the *E. coli ribA* mutant (Figure 2d). Similarly, the plasmid containing *A. salmonicida ribB* rescued the growth of *E. coli* ∆*ribB* riboflavin auxotrophic phenotype without added riboflavin confirming that there is redundancy in RibB activity in *A. salmonicida*, which is mediated by two different proteins (Figure 2e).

**Figure 3.**
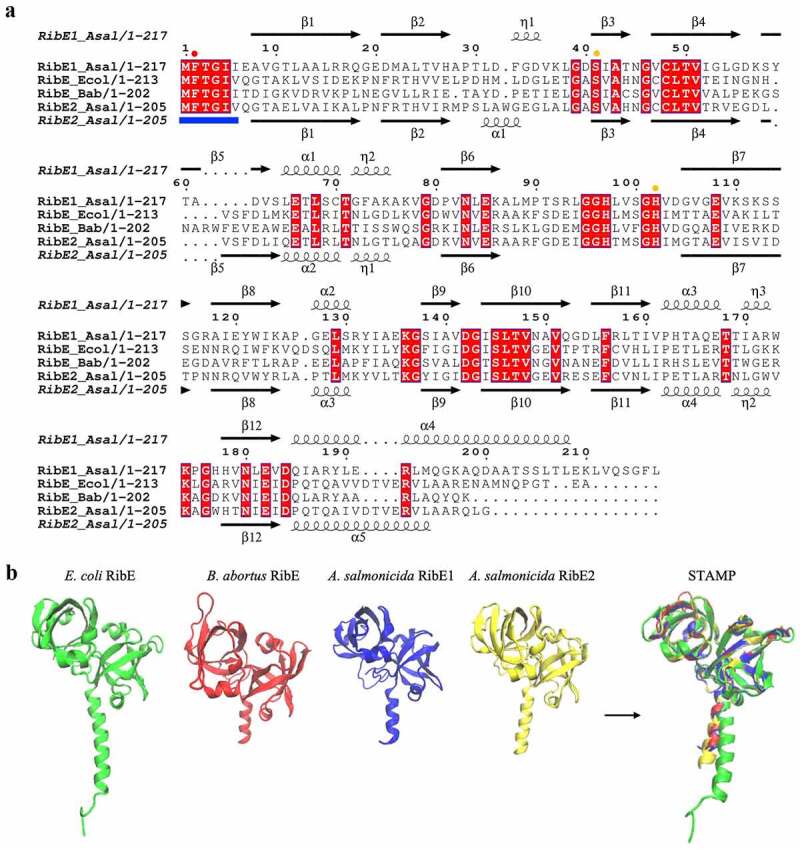
Sequence and structural alignments of *A. salmonicida* riboflavin synthases RibE1 and RibE2. a. Multiple sequence alignment of *E. coli* (*Ecol*), *B. abortus* (*Bab*), and *A. salmonicida* (*Asal*) RibE proteins. The secondary structures at the top and bottom of the alignment correspond to the *A. salmonicida* RibE1 and RibE2, respectively (spirals represent α-helix; arrows represent β-sheet). Conserved amino acid residues are highlighted in red. The circles indicated the one critical residue required for the enzyme activity (Phe-2; red circle) and two other residues required for the significant enzyme activity improvement (Ser-41 and His-102; orange circle). Also, residues that may be involved in substrate recognition (Met-1, Phe-2, Thr-3, Gly-4, Ile-5, and Ile-6/val-6) are also indicated in the amino-terminal section with the blue line in the bottom of the alignment b. Structural alignment of RibE proteins from *E. coli*, *B. abortus*, and *A. salmonicida*. Sequence alignment of riboflavin synthases was performed in JAL view using Clustal Omega Multiple Sequence Alignment Program and rendered using the web-based interface of ESPript. 3D structures of *A. salmonicida* J223 riboflavin synthases were predicted using the trRosetta protein structure prediction service and visualized in VMD. Structural alignments of 3D protein structures of riboflavin synthases were performed using Structural Alignment of Multiple Proteins (STAMP) on VMD.

Another *A. salmonicida* RBP gene showing possible duplication is RibE. To get insights into the functionality of the two putative *A. salmonicida* RibE homologs, RibE1 and RibE2 were aligned to *E. coli* and *Brucella abortus* RibE [80,81]. In this alignment, one critical residue required for the enzyme activity (Phe-2) and two other residues that provide significant enzyme activity improvement (Ser-41 and His-102) are indicated (Figure 3a). Residues that may be involved in substrate recognition (Met-1, Phe-2, Thr-3, Gly-4, Ile-5, and Ile-6/Val-6) are also indicated in the amino-terminal section (Figure 3a). *A. salmonicida* RibE1 and RibE2 share 33.17 % identity. Both *A. salmonicida* RibE1 and RibE2 conserve the residues required for full activity (Phe-2, Ser-41, and His-102). Thus, sequence analysis suggests that *A. salmonicida* RibE1 and RibE2 function as riboflavin synthases.

Despite several attempts, our group could not obtain an *E. coli ribE* null mutant to perform complementation experiments. Thus, to obtain further information on the functionality of these proteins, their structures were predicted using trRosetta and compared to the reported *B. abortus* and *E. coli* RibE structures [80,81]. The predicted structures of RibE1 and RibE2 were highly similar to each other (Figure 3b). RibE1 and RibE2 structures overlapped with a good structural homology (Q_H_ = 0.9552). These structures were also highly similar to *B. abortus* and *E. coli* RibE (Figure 3b). *B. abortus* RibE arranges in trimers, with monomers showing two characteristic six-stranded β-barrels formed by the amino and carboxyl-terminal domains [81]. *A. salmonicida* RibE1 and RibE2 also formed these two β-barrel domains in the *in-silico* modelations (Figure 3b). Therefore, structural models suggested that the *A. salmonicida* RibE1 and RibE2 are similar to a fully characterized RibE protein and likely possess riboflavin synthase activity.

**Figure 4.**
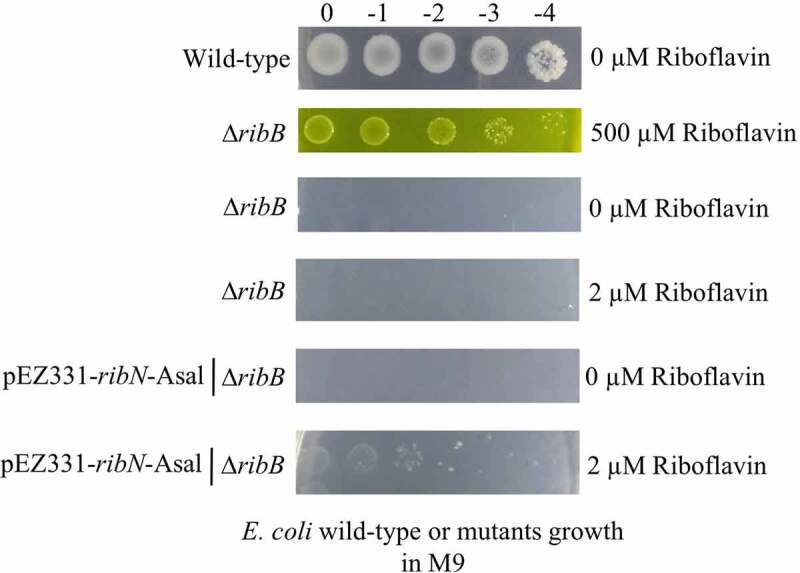
Functionality of *A. salmonicida* RibN family transporter is confirmed by complementing *E. coli ribB* mutant with the plasmid expressing the *A. salmonicida ribN*. Complementation assays were performed in minimal media (M9) plates supplemented with no (0 µm riboflavin), low (2 µm riboflavin), and high (500 µm riboflavin) riboflavin.

The functionality of the putative *A. salmonicida ribN* gene was also assessed by complementing *E. coli* ∆*ribB* in M9 minimal media. *E. coli* ∆*ribB* did not grow in M9 or M9 supplemented with 2 µM of riboflavin, but it grew in M9 supplemented with 500 µM riboflavin (Figure 4). A plasmid expressing *ribN* from *A. salmonicida* rescued the *E. coli* ∆*ribB* phenotype in M9 with low riboflavin (Figure 4), strongly suggesting that RibN functions as a riboflavin importer.

Overall, these results indicate that *A. salmonicida* has a full RBP and a RibN transporter for riboflavin provision. Most of the genes required for the RBP are in the main operon, including *ribD*, *ribE*, *ribBX* and *ribH*. The *ribA* gene and additional copies of *ribB* and *ribE* (*ribE2*) are encoded outside this operon.

### Riboflavin influences the expression of a small set of genes including *ribB*

**Figure 5.**
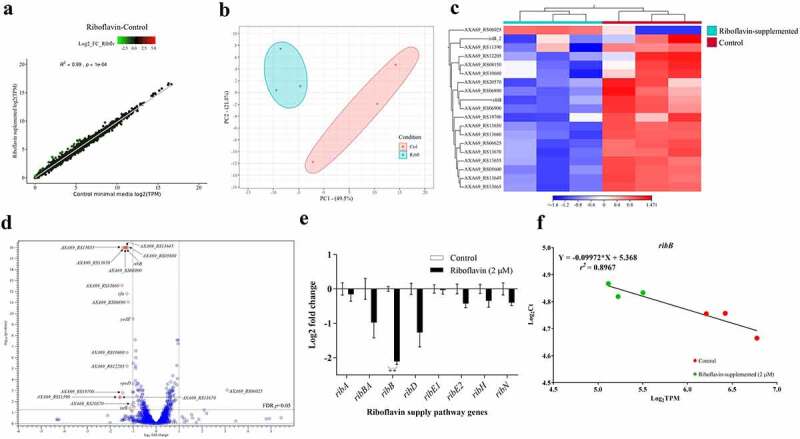
Effect of extracellular riboflavin on *A. salmonicida* J223 global transcriptomic response and expression of riboflavin supply pathway genes. *A. salmonicida* J223 grown in the presence and absence of riboflavin in minimal media. The RNA-Seq experiment involved 6 RNA libraries with three biological replicates for two distinct conditions; control (minimal media) versus riboflavin supplemented (minimal media + riboflavin-2 μM). a. Scatter plot of RNA-seq expression under control and riboflavin supplemented conditions. Red, green, and black dot colours stand for up-, down-, and non-differentially expressed genes, respectively, with each dot representing a gene. b. Principal component analysis (PCA) of *A. salmonicida* samples grown in presence and absence of riboflavin in minimal media, based on the expression of all data sets. c. Hierarchical cluster analysis of RNA-seq results. DEGs are clustered on a heat map; the control (red) and riboflavin-supplemented (aqua) bacterial samples are indicated by the colour bars below the horizontal cluster. d. Volcano plot of DEGs (Cut-off: Log_2_ fold-change (FC) ≥ |1| and false discovery rate (FDR) *p*≤0.05). e. Relative expression of riboflavin supply pathway genes in *A. salmonicida* grown in minimal media with (2 μM) and without riboflavin. Expression of genes *ribA*, *ribB*, *ribBA*, *ribD*, *ribE1*, *ribE2*, *ribH*, and *ribN* in cultures with and without riboflavin was assessed by RT-qPCR. The normalizers were *era* and *hfq*. Asterisks (*) represent the statistically significant differences (***p < 0.01) in the gene expression between control and riboflavin-supplemented *A. salmonicida* cultures, as determined by the non-parametric Kruskal-Wallis test, followed by Dunn’s multiple comparison post-hoc test. f. Correlation between gene expression levels of *ribB* from RT-qPCR and RNA-Seq data. A simple linear regression analysis was performed between the normalized counts (TPM) of RNA-seq data (Log_2_ TPM on the X axis) and the Ct values from RT-qPCR (Log_2_ Ct on the Y axis).

Being a riboflavin-prototroph that can also internalize riboflavin, it is intriguing how external riboflavin affects *A. salmonicida* physiology. To determine the effect of extracellular riboflavin on the genetic expression of *A. salmonicida*, global gene expression profiles of bacteria grown in M9 minimal media and M9 supplemented with 2 µM riboflavin were determined by RNA-seq. Information on sequencing statistics is provided in Table S4. Control and riboflavin-supplemented samples showed a highly significant positive correlation (r^2^ = 0.99; *p* < 0.0001) according to the global expression correlation analysis (Figure 5a). Principal Component Analysis (PCA) and heat map with hierarchical clustering exhibited a clear segregation of control and riboflavin-supplemented samples (Figures 5b,c). A total variation of 71.3% in the expression data was explained by PC1 and PC2 (Figure 5b). The close distribution of samples under riboflavin-supplemented circumstances in comparison to the control samples demonstrates the impact of extracellular riboflavin on gene expression (Figure 5b). For the differential gene expression analysis, a log_2_ FC ≥ |1| and FDR *p*-value of 0.05 were used as the cut-off values. Only 19 genes were differentially expressed by *A. salmonicida* in response to extracellular riboflavin. Of these, 1 gene was upregulated, and 18 were downregulated (Figures 5d and S4A; Table 3; Supplementary file 1). The only upregulated gene encodes for a protein with putative transposase activity. Five of the downregulated genes are found to be part of a cluster in the genome. These genes were *cfa*, coding for a putative cyclopropane-fatty-acyl-phospholipid synthase, and the ORFs A×A69_RS13645 (nuclear transport factor 2 family protein), A×A69_RS13650 (short chain dehydrogenase family NAD(P)-dependent oxidoreductase), A×A69_RS13655 (FAD-dependent oxidoreductase), A×A69_RS13660 (DUF1365 domain-containing protein), and A×A69_RS13670 (DUF2878 domain-containing protein). These genes are adjacent to each other, and an analysis using operon-mapper indicates that they are predicted to form an operon. Although no experimental information is available on the function of this cluster, the presence of *cfa* and other ORFs coding for enzymes involved in RedOx reactions suggests its involvement in fatty acid metabolism [82]. Riboflavin and iron have been proposed to reciprocally regulate their metabolic genes based on their common function as RedOx cofactors [83]. In this case, the A×A69_RS20570 ORF coding for a component of the ABC transport system of the amonabactin siderophore and *yedZ*, coding for a haem-cofactor subunit that works as an electron chain component of the MsrPQ (methionine sulphoxide reductase) system that repairs oxidized periplasmic proteins, were downregulated by external riboflavin (Table 3). Importantly, according to this transcriptomics analysis, the only riboflavin supply gene affected by external riboflavin was the monofunctional *ribB*, while neither any of the rest of the biosynthetic genes nor *ribN* was affected (Table 3). This agrees with the presence of a putative FMN riboswitch in *ribB*. The rest of the genes with a reduced expression included regulators, Lon protease substrate binding-like domain encoding gene, ATPase encoding gene involved in insertion sequences mobility, and mostly genes of unknown function (Table 3). In summary, results indicated that extracellular riboflavin impacts *A. salmonicida* J223 transcriptome response, mainly affecting a few genes probably involved in lipid metabolism, transposition, iron metabolism and one involved in riboflavin supply. To validate the transcriptomics results and corroborate the effects of riboflavin on supply genes, the expression of genes of the RBP and *ribN* in the absence and presence of riboflavin was assessed by RT-qPCR. In accordance with the transcriptomics, a statistically significant two-fold repression of *ribB* expression was detected in RT-qPCR (Figure 5e), and a significant correlation (r^2^ = 0.8967; *p* < 0.05) was observed between *ribB* gene expression levels from RNA-seq and RT-qPCR (Figure 5f). While other RBP genes showed some variability in expression, such differences did not reach statistical significance (Figure 5e), and the correlation between these genes” expression and the RNA-seq data is displayed in Figure S4B.

**Table 3.** Nineteen Differentially Expressed Genes (DEG) from transcriptomics.

Locus Tag	Region	Gene Symbol	Putative Protein Product	Log_2_ Fold Change	FDR p-Value	Conserved Domain	Putative Function
AXA69_RS06025	1259692.1260842	-	IS3 family transposase	3.11	1.0 × 10^−3^	Transpos_IS3	Transposase activity
AXA69_RS06625	Complement (1370582.1371214)	*yedZ*	Sulphoxide reductase haem-binding subunit YedZ	−1.00	3.2 × 10^−10^	Cytochrome_b_N	Haem binding – protect from oxidative stress
AXA69_RS08150	Complement (1707332.1707850)	*rpoD*	Sigma-70 family RNA polymerase sigma factor	−1.01	2.0 × 10^−4^	PRK09651	DNA binding and sigma factor activity
AXA69_RS20570	4389171.4390238	-	Amonabactin ABC transporter permease subunit 1	−1.05	1.9 × 10^−2^	FecCD	Permease of amonabactin siderophore synthesis cluster
AXA69_RS01345	Complement (286176.286931)	*istB*	IS21-like element ISAs29 family helper ATPase	−1.13	4.1 × 10^−2^	YlqF_related_GTPase	ATP binding
AXA69_RS06890	Complement (1431017.1431694)	-	ChrR family anti-sigma-E factor	−1.20	9.3 × 10^−12^	Cupin_RmlC-like	Negative regulation of transcription
AXA69_RS05600	Complement (1172054.1172773)	-	SDR family NAD(P)-dependent oxidoreductase	−1.23	0	NADB_Rossmann	Oxidoreductase activity
AXA69_RS12205	Complement (2534814.2535632)	-	Hypothetical protein	−1.26	6.2 × 10^−6^	N/A	Unknown
AXA69_RS10660	2232777.2233202	-	Hypothetical protein	−1.26	3.6 × 10^−7^	N/A	Unknown
AXA69_RS13665	2851821.2853077	*cfa*	Cyclopropane-fatty-acyl-phospholipid synthase family protein	−1.26	1.6 × 10^−12^	cfa	Methyl transferase in lipid biosynthesis/metabolism
AXA69_RS13645	2848535.2848963	-	Nuclear transport factor 2 family protein	−1.27	0	SnoaL_2	Protein transport into the nucleus and small GTPase binding
AXA69_RS01425	Complement (300198.300851)	*ribB*	3,4-dihydroxy-2-butanone-4-phosphate synthase	−1.32	0	DHBP_synthase	Riboflavin biosynthesis
AXA69_RS06900	Complement (1432389.1432955)	-	LON peptidase substrate-binding domain-containing protein	−1.35	0	LON	ATP-dependent peptidase activity
AXA69_RS13650	2848960.2849694	-	SDR family NAD(P)-dependent oxidoreductase	−1.39	0	NADB_Rossmann	Oxidoreductase activity
AXA69_RS19700	4189195.4189812	-	Hypothetical protein	−1.44	1.5 × 10^−3^	P-loop_NTPase	Unknown
AXA69_RS13655	2849691.2850950	-	FAD-dependent oxidoreductase	−1.45	0	COG2907	FAD binding and oxidoreductase activity
AXA69_RS13660	2850947.2851699	-	DUF1365 domain-containing protein	−1.48	2.8 × 10^−13^	DUF1365	Unknown
AXA69_RS11390	Complement (2375409.2375861)	-	Hypothetical protein	−1.56	4.4 × 10^−3^	N/A	Unknown
AXA69_RS13670	2853080.2853559	-	DUF2878 domain-containing protein	−1.59	3.9 × 10^−3^	DUF2878	Unknown

### Riboflavin biosynthesis genes *ribA*, *ribB*, and *ribE1* are required for *A. salmonicida* virulence in lumpfish

**Figure 6.**
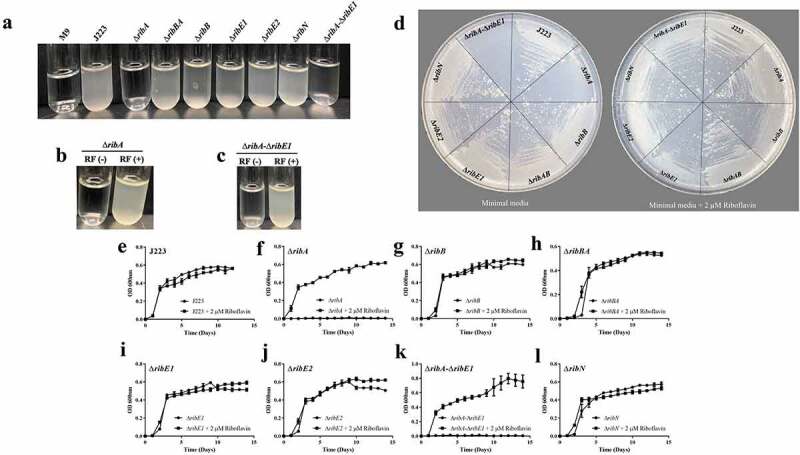
Growth of *A. salmonicida* J223 wild-type and mutant strains in M9 minimal media supplemented with (2 µm) and without riboflavin (RF). a. Growth of J223 and mutants in M9. b. Growth of ∆*ribA* in M9 supplemented without RF(-) and with RF(+) riboflavin. c. Growth of ∆*ribA*-∆*ribE1* in M9 supplemented without RF(-) and with RF(+) riboflavin. d. Growth of *A. salmonicida* J223 and mutants in M9 agar plates with and without riboflavin. Growth curves of e. *A. salmonicida* J223 wild-type, and *A. salmonicida* mutants f. Δ*ribA*, g. Δ*ribB*, h. Δ*ribBA*, i. Δ*ribE1*, j. Δ*ribE2*, k. Δ*ribA-*Δ*ribE1*, and l. Δ*ribN* grown in minimal media in the presence (2 μM) and absence of riboflavin at 15 ºC in triplicates with aeration (180 rpm) for 15 days.

In order to assess whether riboflavin biosynthesis, its duplicated genes and riboflavin uptake are required for virulence in *A. salmonicida*, the single mutant strains Δ*ribA*, Δ*ribB*, Δ*ribBA*, Δ*ribE1*, Δ*ribE2*, and Δ*ribN* were constructed. In addition, a double Δ*ribA*-Δ*ribE1* mutant, combining deletions in a unique and in a duplicating main-operon riboflavin biosynthetic gene was obtained. As an initial characterization, the growth of *A. salmonicida* J223 wild-type and its derivative mutants in minimal media in the presence and absence of 2 µM riboflavin was assessed. *A. salmonicida* wild-type and mutants Δ*ribB*, Δ*ribBA*, Δ*ribE1*, Δ*ribE2*, and Δ*ribN* grew in the presence and absence of riboflavin with no significant differences (Figures 6a–l). In contrast, Δ*ribA* and Δ*ribA*-Δ*ribE1* mutants did not grow in M9 minimal media without riboflavin, and growth was restored by supplementing the M9 with a low concentration of riboflavin, indicating that they are riboflavin auxotrophs (Figures 6b–d). The riboflavin auxotrophic phenotype of these mutants was confirmed by growth curves, where significantly higher growth of Δ*ribA* and Δ*ribA*-Δ*ribE1* was observed in the presence of riboflavin (Figures 6F–k). Expectedly, the Δ*ribN* mutant has no growth defect in either condition as endogenous biosynthesis supplies the vitamin (Figure 6l). The requirement of external riboflavin for the growth of the Δ*ribA* strain confirmed that the RibBA protein does not display RibA activity and that the independent *ribA* codes for the only GTP cyclohydrolase II in *A. salmonicida* (Figure 6F). Endogenous riboflavin provision is not compromised in the Δ*ribB* as the RibBA maintains RibB activity (Figure 6g). In the same way, the Δ*ribE1* strain not becoming riboflavin auxotroph supports the notion of that *ribE2* codes for a functional riboflavin synthase, and likewise, in the Δ*ribE2* mutant the biosynthesis would be sustained by *ribE1* (Figures 6i–j).

A biochemical profile analysis using the API 20E and API 20NE showed few differences between the wild-type and mutants. *A. salmonicida* Δ*ribA-*Δ*ribE1* displayed a negative reaction for the hydrolysis of L-arginine, while others displayed a positive reaction (Tables S5 and S6). The *A. salmonicida* Δ*ribB* mutant showed a negative reaction for the naphthol-AS-BI-phosphohydrolase enzyme assay, while others showed positive reactions (Table S7).

**Figure 7.**
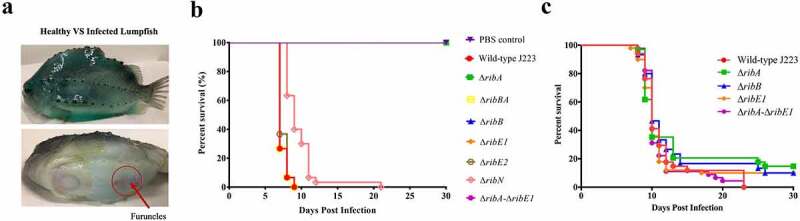
Virulence and immune protection of *A. salmonicida* mutants in lumpfish. **A**. Infected lumpfish showed furunculosis clinical signs compared to healthy fish. **B**. Lumpfish survival (%) after ip infection with 10^4^ CFU/dose of *A. salmonicida* wild-type J223 and mutants. No significant difference was detected between PBS control, mutants Δ*ribA*, Δ*ribB*, Δ*ribE1*, and Δ*ribA*-Δ*ribE1*. However, wild-type and mutants Δ*ribBA*, Δ*ribE2*, and Δ*ribN* infected fish groups showed significantly (*p* < 0.0001) lower survival compared to PBS control and mutants Δ*ribA*, Δ*ribB*, Δ*ribE1*, and Δ*ribA*-Δ*ribE1* infected fish groups. **C**. Survival (%) of lumpfish survivors from attenuated mutants; Δ*ribA*, Δ*ribB*, Δ*ribE1*, and Δ*ribA*-Δ*ribE1* infected groups, after ip challenge with 10^3^ CFU/dose of wild-type *A. salmonicida*. These mutants did not significantly differ in their survival rates from one another. Kaplan-Meier estimator and Log-rank test were used to obtain survival fractions after the infection and to compare survival curve trends, respectively.

**Figure 8.**
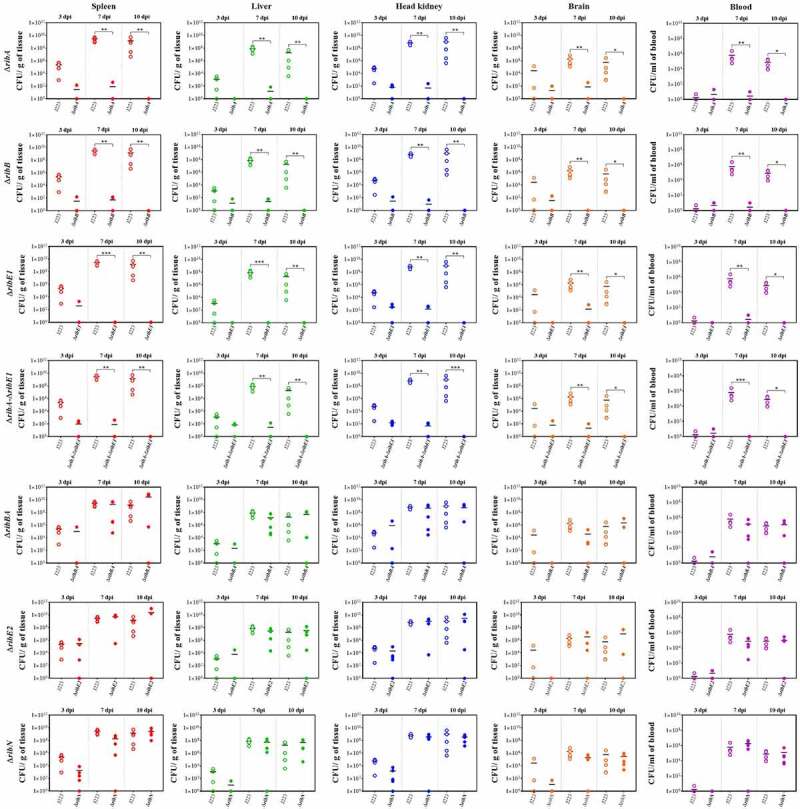
Lumpfish spleen, liver, head kidney, brain, and blood tissue colonization by *A. salmonicida* J223 wild-type versus mutant strains Δ*ribA*, Δ*ribB*, Δ*ribE1*, Δ*ribA-*Δ*ribE1*, Δ*ribBA*, Δ*ribE2*, and Δ*ribN* at 3, 7, and 10 days post-infection. Five fish were sampled from each *A. salmonicida* strain infected fish group at each time point. Asterisks (*) represent the significant differences (*p < 0.05, **p < 0.01, ***p < 0.001) in the tissue colonization between wild type and each mutant strain per time point (3, 7, and 10 dpi), as determined by the non-parametric Kruskal-Wallis test, followed by Dunn’s multiple comparison post-hoc test.

The virulence of *A. salmonicida* J223 wild-type and the mutants was evaluated in lumpfish using an intraperitoneal infection model [63]. Lumpfish were infected with 10^4^ CFU/dose of wild-type or mutants, and their survival was recorded daily. Infected lumpfish showed classic clinical signs of furunculosis, including typical furuncles on the ventral part of the body (Figure 7a). All fish infected with the wild-type, the Δ*ribBA* or the Δ*ribE2* strains died within 10 dpi (Figure 7b). Lumpfish infected with *A. salmonicida* Δ*ribN* showed delayed mortality, reaching 100% after 21 dpi (Figure 7b). In contrast, the Δ*ribA*, Δ*ribB*, Δ*ribE1*, and Δ*ribA-*Δ*ribE1* mutant strains were fully attenuated as fish infected with these strains showed 100% survival (Figure 7b). The bacterial colonization of the spleen, liver, head kidney, brain, and blood was evaluated at 3, 7, and 10 dpi. In agreement with the survival levels, mutants Δ*ribA*, Δ*ribB*, Δ*ribE1*, and Δ*ribA*-Δ*ribE1* showed significantly lower levels of colonization than the *A. salmonicida* J223 wild-type (Figure 8). In contrast, the Δ*ribBA*, Δ*ribE2*, and Δ*ribN* mutants colonized tissues and blood at similar levels to the wild-type (Figure 8). In general, some of these attenuated mutants reached low levels of colonization in organs like the spleen, liver, and head kidney by 3 dpi but they were fully cleared from fish by 10 dpi (Figure 8).

To determine whether the infection with the attenuated mutants could confer immune protection to the lumpfish and to examine the mutants’ utility as live attenuated vaccine, surviving fish from Δ*ribA*, Δ*ribB*, Δ*ribE1*, and Δ*ribA*-Δ*ribE1* infected groups were challenged with 10^3^ CFU/dose (10 times the reported LD_50_ [63]) of *A. salmonicida* J223 wild-type after 30 dpi. All attenuated *A. salmonicida* mutants conferred low levels of protection, producing survival percentages ranging from 10 to 15 % (Figure 7C).

## Discussion

The results of this study show that *A. salmonicida* possesses both riboflavin biosynthesis and uptake functions by having an RBP and a RibN transporter, respectively. The coexistence of RBP and riboflavin transporters is relatively common in bacteria, like *Bacillus subtilis* and *Lactococcus lactis* [23,25,84,85], and seems to be conserved in fish pathogens like *A. salmonicida*.

The riboflavin provision genes landscape is highly variable among bacteria, with duplications or multiplications of functions present in many species. The initial search in *A. salmonicida* suggested that the main RBP operon included a *ribBA* gene coding for a bifunctional enzyme (Figure 1b). Nonetheless, alignments and functional complementation analysis further showed that its product conserves only RibB activity (Figures 2a–c). In other bacteria where similar genes for RibBX fusions have been identified, it has been reported that the putative RibA domain lacks critical residues for zinc binding and ring opening, which are essential for GTP cyclohydrolase II activity [78,79]. Functional complementation, sequence analysis, and phenotypic characterization of mutants revealed that conserved functional duplications are encoded by *ribB* and *ribE2* outside the main operon. In this regard, *A. salmonicida* riboflavin provision pathways are similar to those present in *Shewanella oneidensis* [78]. Notably, the X domain of *S. oneidensis* RibBX fusion lacks GTP-cyclohydrolase II activity, yet it regulates the activity of the associated N-terminal RibB domain by an unknown mechanism [78]. It has been hypothesized that *S. oneidensis* RibBX might have evolved when an alternative gene encoding a GTP cyclohydrolase II was acquired, releasing the original RibBA protein from the selective pressure to keep its GTP cyclohydrolase II activity. In *A. salmonicida*, the GTP cyclohydrolase II activity is provided solely by the monocistronic *ribA* as confirmed by the fact that a null mutation in this gene results in riboflavin auxotrophy, which suggests that the selective pressure to maintain the GTP cyclohydrolase II activity of the *A. salmonicida* RibBA protein may have been removed when this unique *ribA* gene was acquired.

Riboflavin synthase, which is encoded by *ribC* or *ribE* genes, catalyzes the last step of riboflavin biosynthesis (i.e. a dismutation reaction from 6,7-dimethyl-8-ribityllumazine to riboflavin) [2]. Since animals lack this enzyme and the majority of the pathogenic bacteria strictly rely on endogenous riboflavin biosynthesis, riboflavin synthase could be an interesting target for antimicrobial inhibitors, which may cause bacterial riboflavin auxotrophy or reduced virulence without putting the host at risk [7,86]. *A. salmonicida* encodes 2 *ribE* genes; *ribE1* is in the main RBP operon while *ribE2* is encoded outside of the main operon as a monocistronic unit (Figures 1b–e), similar to *S. oneidensis* and *Pseudomonas putida* [78]. Extracellular riboflavin had no significant effect on the expression of *ribE1* and *ribE2* in RNA-seq and qPCR (Supplementary file 1 and Figure 5e). Moreover, *A. salmonicida* Δ*ribE1* and Δ*ribE2* grew similarly to wild-type. Thus, it is possible that *ribE1* and *ribE2* are functionally equivalent and interchangeable when cultured in liquid media. *A. salmonicida* RibE1 and RibE2 monomers are predicted to fold very similarly with a good structural homology (Q_H_ = 0.7219) despite sharing only 33% of protein sequence identity.

Conservation of these highly similar riboflavin synthases with the same function in *A. salmonicida* is intriguing. However, we anticipate that the two *ribE* genes might be differentially expressed inside the host. Strikingly, despite being functionally interchangeable *in vitro*, the Δ*ribE1* strain was fully attenuated while the Δ*ribE2* remained virulent in lumpfish host (Figure 7b and 8). This indicates that *ribE1* is essential for *A. salmonicida* virulence and the provision of riboflavin within the host, while *ribE2* could be a redundant gene copy during host colonization but specifically required to grow in different yet unknown conditions. Additionally, the capacity of *ribE1* to compensate for the absence of *ribE2* may contribute to the virulence of *A. salmonicida* Δ*ribE2*. Differential expression of these two genes inside the host cells may explain this effect. Similarly, differential effects of the two *B. abortus ribH* genes coding for lumazine synthases was demonstrated by Bonomi et al. (2010). In *B. abortus* either *ribH* is sufficient for *in vitro* growth, whereas *ribH2* is specifically required for intracellular proliferation and survival in murine macrophages [7]. It appears that vertical or horizontal gene transfer may cause the duplication of riboflavin synthases or lumazine synthases, which could provide positive fitness to bacteria in terms of virulence, pathogenicity, or other specific conditions. Moreover, results indicated that the extra copies of RBP genes in the genome seem not to be redundant.

Bacterial genomes encode riboflavin transporter proteins in addition to or in substitution of riboflavin biosynthetic genes [11,18]. *A. salmonicida* has a RibN transporter similar to those reported in *V. cholerae*, *Rhizobium leguminosarum*, and other proteobacteria [18,21], located independently from the main riboflavin biosynthesis genes. Previous studies have employed riboflavin auxotrophic strains to evaluate the functionality of riboflavin transporters, like RibN from *Rhizobium leguminosarum* [18] and RibM from *Streptomyces davawensis* [87] by growth complementation in low riboflavin concentration. Similar to these described approaches, in this study, the growth of *E. coli ribB* mutant was rescued in M9 minimal media supplemented with 2 µM riboflavin by the heterologous expression of plasmid carrying *A. salmonicida ribN* (Figure 4), indicating the functionality of *A. salmonicida ribN* as riboflavin transporter. Extracellular riboflavin does not affect *A. salmonicida ribN* gene expression (Figure 5e). Similarly, *R. leguminosarum* and *V. cholerae* showed no differences in the *ribN* expression levels in the presence or absence of riboflavin [18,21]. The growth rate of *A. salmonicida* Δ*ribN* was identical to the wild-type, and no significant growth difference was observed in Δ*ribN* cultured with and without exogenous riboflavin (Figures 6e,l), similar to what was reported in *R. leguminosarum* [18]. Also, Δ*ribN* mutant remained virulent in the lumpfish host, although it caused delayed mortality (Figure 7b). The *A. salmonicida ribN* mutant may have enough riboflavin supply via endogenous biosynthesis when grown in the absence of riboflavin in media, and when it is inside the host. This could explain why no differences in growth rates were noticed (Figure 6l), and why the Δ*ribN* retained virulence (Figure 7b and 8). On the contrary, Garcia-Angulo et al. (2013) demonstrated that the RibN transporter of *R. leguminosarum* is required to enhance colonization of the pea plant nodules [18]. Thus, the biological role of the RibN transporter may vary depending on the physiology of bacterial species.

This study assessed the response induced by the availability of riboflavin in *A. salmonicida* J223 grown in M9 minimal media. Our results showed that the extracellular riboflavin has a moderate impact on gene expression in *A. salmonicida*. Transcriptomics analysis revealed that only 19 genes were differentially expressed (log_2_ FC ≥ |1| and FDR *p* ≤ 0.05) in response to extracellular riboflavin (Table 3, Figures 5c,d), suggesting that exogenous riboflavin is involved in very defined physiological functions. Concurrently, DEG analysis and RT-qPCR results showed that extracellular riboflavin downregulated the monocistronically encoded *ribB* while having no significant effect on the expression of genes in the main RBP operon (*ribH*, *ribBA*, *ribE1*, *ribD*) on which the other *ribB* homolog (i.e. *ribBA*) was encoded (Table 3; Figure 5e). Similar results were observed in *V. cholerae* N16961 cultured in T minimal media without and with riboflavin (2 µM) in a transcriptomic-based approach [83], and in an RT-qPCR analysis [21]. Therefore, in the presence of exogenous riboflavin, *A. salmonicida* might still be able to display the riboflavin biosynthesis function.

In *V. cholerae* transcriptomic analysis (cut-off values of 1 fold change in expression and *p* < 0.05) performed by Cisternas et al. (2018), the number of genes affected by the elimination of riboflavin biosynthesis (*ribD* deletion, 142 DEGs) was substantially greater than the number of genes impacted by the presence of exogenous riboflavin (wild-type grown with 2 µM riboflavin, 26 DEGs) or the elimination of riboflavin transport (*ribN* deletion, 71 DEGs) [83]. Interestingly, the number of genes impacted by extracellular riboflavin in wild-type *V. cholerae* is quite low (i.e. 26) and is comparable to our results (i.e. 19), which suggests the presence of external riboflavin affects only a small number of genes. Overall, findings from our transcriptomic study and that of Cisternas et al. (2018) suggest that the biosynthesis of riboflavin is more relevant for physiological functions than exogenous riboflavin [83].

The only upregulated ORF (AXA69_RS06025) in response to extracellular riboflavin in *A. salmonicida* encodes an IS3 family transposase (Table 3). DNA transposases are enzymes that transfer discrete DNA segments known as transposons from one region of the genome to another region and are typically encoded by the mobile genetic element (i.e. insertion sequences; ISs) [88,89]. The *A. salmonicida* genome is rich in ISs, and ISs-mediated rearrangement events could cause a loss in the *A. salmonicida* virulence [90]. Concomitantly, the effect of ISs in the bacterial genome leads to “genomic plasticity,” which could aid in bacterial adaptation to changing environments, functional virulence, and acquisition of new metabolic capabilities [90–92]. Although the effect of the induction of this gene is not yet evident, it is interesting to investigate the probable role of riboflavin in the induction of genomic plasticity.

Riboflavin availability affects iron metabolism in bacteria, and there is a crucial regulatory crosstalk between these two important RedOx cofactors in many other species [3,83]. In line with this, exogenous riboflavin affected the expression of iron metabolism-related genes that are involved in haem binding (AXA69_RS06625) and siderophore synthesis (AXA69_RS20570) in *A. salmonicida* (Table 3). *A. salmonicida* produces siderophores such as acinetobactin and amonabactin under iron-limited conditions as one of its iron acquisition strategies [93]. Interestingly, amonabactin ABC transporter permease subunit 1, one of the genes in the gene cluster responsible for amonabactin synthesis and transport in *A. salmonicida*, was downregulated in response to riboflavin (Table 3) [93]. In contrast, the expression of other genes in this cluster, including the amonabactin ABC transporter permease subunit 2 was not affected by exogenous riboflavin [93].

The coexistence of *ribN* and a RBP with extra gene copies for *ribB* and *ribE* in *A. salmonicida* is intriguing (Figures 1B–f). The combined presence of functional duplications and transporter function may suggest that these individual biosynthetic and uptake genes are differentially regulated, presumably in response to the demand for flavins that serve purposes distinct from nutritional requirements. For instance, flavins are involved in bacterial virulence [7]. It has been hypothesized before that RBP genes that have been duplicated or multiplicated could have specific functions and provide adaptive benefits to the bacteria [11,28,29]. Thus, the role of the RBP genes and their additional copies, and of the *ribN* transporter in the virulence and physiology of *A. salmonicida* is a question of biological relevance. To get insights into this, we constructed (Figure S5) and characterized (Figure 6; Tables S5-S7) Δ*ribA*, Δ*ribB*, Δ*ribBA*, Δ*ribE1*, Δ*ribE2*, Δ*ribN*, and Δ*ribA*-Δ*ribE1* mutants and then examined their virulence in lumpfish infection model (Figure 7 and 8), which is a well-established marine teleost model to investigate bacterial pathogenesis [41,71,94,95]. This allowed us not only to determine the effects of the different RBP or transporter gene mutations on virulence but also to test the use of the mutants as live attenuated vaccine candidates for lumpfish.

Mutations in critical biosynthetic pathways (i.e. aromatic amino acids, purine, thymine, and riboflavin) of a pathogenic bacterium are known to attenuate and limit the growth or virulence of the pathogen *in vivo* [96]. When the riboflavin biosynthesis operon/gene of two mammal pathogens, *Rhodococcus equi* (i.e. Δ*ribBA*) and *Actinobacillus pleuropneumoniae* (i.e. Δ*ribGBAH*), were disrupted, both of these mutants became avirulent, making them potential live-attenuated vaccine candidates [97,98]. *A. salmonicida* Δ*aroA* mutants are attenuated because they lack biosynthesis of *p*-aminobenzoic acid, which is essential for folate (vitamin B9) synthesis, and so this strain has been used as a vaccine in Atlantic salmon [99]. In our study, *A. salmonicida* mutants Δ*ribA*, Δ*ribB*, Δ*ribE1*, and Δ*ribA*-Δ*ribE1* were fully attenuated. Their colonization began at 3 dpi, it was significantly low at 7 dpi compared to the wild-type and the other virulent mutants, and then bacteria were cleared from tissues and blood at 10 dpi (Figure 8). Therefore, it is evident that the attenuation facilitated host immune clearance. *A. salmonicida* attenuated mutants could not establish a systemic infection and extensive proliferation in lumpfish probably due to the limited availability of riboflavin in the fish host milieu. Also, it appeared that *A. salmonicida ribA*, *ribB*, and *ribE1* genes are essential for the riboflavin supply during host colonization and influence virulence. Overall, riboflavin uptake cannot compensate for biosynthesis during infection; hence riboflavin biosynthesis is essential for *A. salmonicida* virulence and physiology. We next questioned whether the attenuated mutant strains retained immunogenicity and provided protection to lumpfish. However, after challenging the immunized lumpfish with the wild-type, we observed that these mutants confer only modest immune protection with low RPS (~10 to 15%) (Figure 7C). The lack of rounds of mutants’ replication within the fish host may be a feasible explanation for why the *ribA*, *ribB*, *ribE1* and *ribA-ribE1* mutants do not provide sufficient immune protection. In other words, mutants are simply too attenuated to adequately colonize at the appropriate time or in sufficient numbers to trigger a proper and protective memory immune response. Therefore, *A. salmonicida* riboflavin auxotrophic mutants of RBP (i.e. Δ*ribA*, and Δ*ribA*-Δ*ribE1*) may not be useful in live-attenuated vaccine design against this pathogen due to their hyper-attenuation. On the other hand, the profound immune suppression imposed on lumpfish by *A. salmonicida* J223 strain may preclude protective immunity [63]. Further studies are required to improve the immunogenicity of the attenuated mutant strains, for instance, using a regulated-delayed attenuation strategy [100] or the overexpression of protective immunogenic antigens by these strains.

## Conclusion

This study is the first report of riboflavin supply pathways in a marine fish bacterial pathogen, *A. salmonicida*, and comprises integral analyses investigating the host-pathogen-riboflavin interactions. Our results indicate that *A. salmonicida* has an RBP with extra gene copies for *ribB* and *ribE*, and *ribN* family transporter, which are encoded in five transcriptional units. Exogenous riboflavin affects the transcriptome response and differentially regulates the expression of riboflavin supply genes. Mutations in *ribA*, *ribB* and *ribE1* have an impact on bacterial virulence, host colonization and immune protection. The *ribE2* gene is redundant during lumpfish host colonization. In summary, we showed that riboflavin biosynthesis is essential for *A. salmonicida* virulence and physiology during lumpfish infection.

## Supplementary Material

Supplemental Material

## Data Availability

The datasets presented in this study can be found in online repositories. The raw sequence data are deposited in the NCBI Sequence Read Archive (SRA) with the accession number PRJNA909183. The name of the repository and accession number(s) can be found at: https://www.ncbi.nlm.nih.gov/bioproject/PRJNA909183.
