# Supplementary material for: Role of riboflavin biosynthesis gene duplication and transporter in Aeromonas salmonicida virulence in marine teleost fish
Source: Virulence. 2023 Mar 9;14(1):2187025. doi: 10.1080/21505594.2023.2187025 (PMC10012899; doi:10.1080/21505594.2023.2187025)
Supplement: Supplemental Material [file KVIR_A_2187025_SM7317.zip › Supplementary-tables-RF-Virulence-2023.docx]

**Table S1**. Primers for Reverse Transcription PCR (RT-PCR) used to amplify gene junctions of *rib* gene operons

| Primer name | Sequence (5’ to 3’) | Gene junction | Amplicon size (bp) |
| --- | --- | --- | --- |
| cysB-F | CAGCCAGCAACTGACACAAGGT | *cysB - ribA* | 400 |
| ribA-R | GAAAGCCTACCAGCGTGAA |  |  |
| ribA-F | CCTCTCGACCAAAGCGAATAA | *ribA - cmk* | 228 |
| cmk-R | TGCCATGTTTGCCGATATAGAG |  |  |
| 3030-F | ATTGTTGCGCTGATCGAAGC | *nrdR* - AXA69_003030 | 246 |
| nrDR-R | TATCAACCGCACTACAGAAAGG |  |  |
| nrDR-F | ATGGATGAGCTCAAGAGCCTG | *ribD* - *nrdR* | 413 |
| ribD-R | CAGGGTCACATAAGCGGTAG |  |  |
| ribD-F | ATCAAGGATGTGCGGCTGGTGG | *ribE1* - *ribD* | 144 |
| ribE1-R | GTTCCCACCGCTTCGATAAT |  |  |
| ribE1-F | GTTATCTGGAGCGGCTGATG | *ribBA - ribE1* | 304 |
| ribBA-R | ATCCATCAGGATCACCATCTT |  |  |
| ribBA-F | GTGGAATACGTCGGCGAATAA | *ribH - ribBA* | 248 |
| ribH-R | GTCCACCAAGCTTTCGTTGAT |  |  |
| ribH-F | TACCAAGGCGGGTAACAAGG | *nusB - ribH* | 254 |
| nusB-R | AACAGCAGATCGCGGAAATA |  |  |
| nusB-F | ACTGGACAAGGTGATCAAGAC | *thiL - nusB* | 360 |
| thiL-R | GGCGAGATCGGACAAATTGA |  |  |
| 8895-F | CGATGCGAATGGCCTCACAA | AXA69_018895 *- ribE2* | 244 |
| ribE2-R | ATCACATGGGTGCGAAAGT |  |  |
| ribE2-F | AACCTGGGTTGGGTCAAAG | *ribE2 -* AXA69_018885 | 440 |
| 8885-R | ATCCCTCGGTGAGTTGAATG |  |  |
| purU-F | TGTTGAGCTGGTGCTTGTAG | *purU - ribB* | 600 |
| ribB-R | GGGATCACCAAATTCACTGAGTA |  |  |
| ribB-F | TGAGCTGACCAAAGAAGATGG | *ribB -* AXA69_001415 | 439 |
| 1415-R | TTCCAGTTCGCTGGTGTTT |  |  |
| 8915-F | AGCCAGGAAGCCGTCTAT | AXA69_018915 *- ribN* | 403 |
| ribN-R | ATATTGGCTCAGACTGTTGAC |  |  |
| ribN-F | CTGGCTGGCTGGTGTTT | *ribN - purT* | 367 |
| purT-R | TTGTGTGCCACCTGCAT |  |  |

**Table S2.** qPCR primers and primer efficiency

| **Gene** | **Forward primer (5’ to 3’)** | **Tm (^o^C)** | **Reverse primer (5’ to 3’)** | **Tm (^o^C)** | **Amplicon length (bp)** | **% Efficiency** |
| --- | --- | --- | --- | --- | --- | --- |
| *ribA* | CTGCGCCTGATGACCAATAA | 55 | GGTAGAACTCGTTGTGAGGATTG | 55 | 115 | 98.85 |
| *ribB* | CATGATGATCCGCGAGTGTT | 55 | GTCTGATAGTGGCTGGAGTTTG | 55 | 111 | 91.45 |
| *ribBA* | AAACTGCCCACCGAGTTT | 53 | GGTCTGGATGTCACCTTTCTT | 54 | 99 | 90.38 |
| *ribE1* | GAATCTCGAGGTGGATCAGATAG | 53 | GACCAGTTTCTCCAGAGTCAG | 54 | 100 | 94.91 |
| *ribD* | GCCAAGCGGAACATCAATTC | 54 | GATAAAGCACCAGCTCATCCA | 54 | 97 | 89.38 |
| *ribE2* | GTGAAGTGCGAGAGAGTGAAT | 54 | GGATCGATCTCGATATTGGTGTG | 55 | 109 | 88.91 |
| *ribH* | CAAGTCCAGGACAGCAATCT | 54 | TGGCATCGTATTGACCACTC | 54 | 100 | 90.70 |
| *ribN* | CCAGGTTGCCTTTCATCAATAC | 54 | CGCAATTGGTTGGTCATCAG | 54 | 99 | 104.84 |

| Primer | Sequence (5’ to 3’) |
| --- | --- |
| ∆*ribBA A.sal* (*SphI*) F1 | ACATGCATGCGGTGGGTGAGGTGAAGAGCAAGTCC |
| ∆*ribBA A.sal* (*XhoI-PstI*) R1 | CTCGAGCGGAAAACTGCAGTTTTAGAGCGGGATCACAGAAAATCGTTT |
| ∆*ribBA A.sal* (*XhoI-PstI*) F2 | AAAACTGCAGTTTTCCGCTCGAGTAAGCACGTCGCCCGCATGACATTG |
| ∆*ribBA A.sal* (*XbaI*) R2 | TCGTCTAGAGTGCCGCCACGGATGACGGTGCCCA |
| ∆*ribE1 A.sal* (*SphI*) F1 | ACATGCATGCACAAGGCGGCTGGCGACTGGCCTGA |
| ∆*ribE1 A.sal* (*XhoI-PstI*) R1 | CTCGAGCGGAAAACTGCAGTTTTGCAGATCACCTTGATGATGACGGGG |
| ∆*ribE1 A.sal* (*XhoI-PstI*) F2 | AAAACTGCAGTTTTCCGCTCGAGAACATAATTCTGTATCAAAGTGAGC |
| ∆*ribE1 A.sal* (*XbaI*) R2 | TCGTCTAGATGGCATCGGGCGCCACGGCGGCCTG |
| ∆*ribE2 A.sal* (*SphI*) F1 | ACATGCATGCGGAGACAAAATGCGGGGGTGTCATT |
| ∆*ribE2 A.sal* (*XhoI-PstI*) R1 | CTCGAGCGGAAAACTGCAGTTTTGTCTCTTCTCCTGCTACGAAAAAAGGC |
| ∆*ribE2 A.sal* (*XhoI-PstI*) F2 | AAAACTGCAGTTTTCCGCTCGAGGGTCAAAGCCGGTTGGCACACCAATAT |
| ∆*ribE2 A.sal* (*XbaI*) R2 | TCGTCTAGAGCTCAGATCCCTCGGTGAGTTGAAT |
| ∆*ribA A.sal* (*SphI*) F1 | ACATGCATGCTCGAGTTCCACATCATGGTGCAAGG |
| ∆*ribA A.sal* (*XhoI-PstI*) R1 | CTCGAGCGGAAAACTGCAGTTTTAGGTTGCTCCTTACTACAGCTGCTT |
| ∆*ribA A.sal* (*XhoI-PstI*) F2 | AAAACTGCAGTTTTCCGCTCGAGTGAGCCTCACAACGAGGCCTGCCTTT |
| ∆*ribA A.sal* (*XbaI*) R2 | TCGTCTAGACAAAACCTATGTTCGACTGGGCGTC |
| ∆*ribB A.sal* (*SphI*) F1 | ACATGCATGCCCATCTCTGTCATGATTCCGTTTGT |
| ∆*ribB A.sal* (*XhoI-PstI*) R1 | CTCGAGCGGAAAACTGCAGTTTTGCAATATCTGTCTTATCCTCTTTCATC |
| ∆*ribB A.sal* (*XhoI-PstI*) F2 | AAAACTGCAGTTTTCCGCTCGAGTGAGCCTCATACGAAATTGTAATAAG |
| ∆*ribB A.sal* (*XbaI*) R2 | TCGTCTAGATGCAACATCAGCAGCAATCTTGGAT |
| ∆*ribN A.sal* (*SphI*) F1 | ACATGCATGCACGTTCGTTCAGGTTGTCACCACCG |
| ∆*ribN A.sal* (*XhoI-PstI*) R1 | CTCGAGCGGAAAACTGCAGTTTTGGGATCCGGGTCGCTGGAAAAAAGT |
| ∆*ribN A.sal* (*XhoI-PstI*) F2 | AAAACTGCAGTTTTCCGCTCGAGTGAGGTACGCCATGCGTGCCTCTTT |
| ∆*ribN A.sal* (*XbaI*) R2 | TCGTCTAGA CTTGCTCAAGCTGTGCGAGGGTATC |
| P_lac_-*ribBA* F | TTTACACTTTATGCTTCCGGCTCGTATGTTATGGCGCTGAGCACAACCCAGGAAA |
| *ribBA* R | TTATTCGCCGACGTATTCCACCACT |
| P_lac_-*ribE1* F | TTTACACTTTATGCTTCCGGCTCGTATGTTATGTTTACCGGAATTATCGAAGCGG |
| *ribE1* R | TTACAGAAAGCCGGACTGGACCAGT |
| P_lac_-*ribE2* F | TTTACACTTTATGCTTCCGGCTCGTATGTTATGTTCACCGGCATAGTGCAGGGGA |
| *ribE* R | GATCAGGTTGACGCAAAATTCACTC |
| P_lac_-*ribA* F | TTTACACTTTATGCTTCCGGCTCGTATGTTATGAGCAGCGTTACCCTCGTGGCCA |
| *ribA* R | TCACTTCTTGAACATGTGGTCCAAT |
| P_lac_-*ribB* F | TTTACACTTTATGCTTCCGGCTCGTATGTTATGAATCAGTCTCTACTCAGTGAAT |
| *ribB* R | TCAGGCAGAACGTTCTGACAGCAAT |
| P_lac_-*ribN* F | TTTACACTTTATGCTTCCGGCTCGTATGTTATGGCACCCTCGCGCAGGCACTCTC |
| *ribN* R | TCAGGAAACAGATTGATGGGCGCGC |
| *E. coli*-*ribA*-H1P1 | TATCTGGAGAATTTCATGCAGCTTAAACGTGTGGCAGAATGTAGGCTGGAGCTGCTTCG |
| *E. coli*-*ribA*-H2P2 | AGCAAATGAATTACACAATGCAAGAGGGTTATTTGTTCACATATGAATATCCTCCTTAG |
| *E. coli*-RibA-Fw | ATTCTCGAGGCAATCGAACGCATGGCCTCTCC |
| *E. coli*-RibA-Rv | ATTAAGCTTTATGTTGAAGTAACAACTATTTGC |

**Table S3.** Primers for mutants and plasmids construction

**Table S4.** Mapping Statistics of Transcriptomics

| Experimental condition | Number of reads | Number of reads after trimming | Percentage (%) trimmed | Mapped reads | Percentage (%) reads mapped |
| --- | --- | --- | --- | --- | --- |
| ASAL_Control_1 | 138,818,018 | 138,757,015 | 99.96 | 137,107,214 | 98.85 |
| ASAL_Control_2 | 124,141,028 | 124,075,517 | 99.95 | 122,783,010 | 99.00 |
| ASAL_Control_3 | 125,508,488 | 125,396,689 | 99.91 | 123,806,818 | 98.81 |
| ASAL_Riboflavin_1 | 121,754,142 | 121,658,530 | 99.92 | 120,190,512 | 98.86 |
| ASAL_ Riboflavin_2 | 172,517,820 | 172,340,374 | 99.90 | 170,258,416 | 98.89 |
| ASAL_ Riboflavin_3 | 115,788,490 | 115,727,832 | 99.95 | 114,385,936 | 98.89 |

**Table S5.** Biochemical profiles of wild-type and mutants of *A. salmonicida* using the API 20E

| **Enzyme Assayed for** | **J223** | ***∆ribA*** | ***∆ribAB*** | ***∆ribB*** | ***∆ribE1*** | ***∆ribE2*** | ***∆ribN*** | ***∆ribA*-*∆ribE1*** |
| --- | --- | --- | --- | --- | --- | --- | --- | --- |
| β-Galactosidase | - | - | - | - | - | - | - | - |
| Indole production | - | - | - | - | - | - | - | - |
| Acetoin production | + | + | + | + | + | + | + | + |
| Citrate utilization | - | - | - | - | - | - | - | - |
| H_2_S production | - | - | - | - | - | - | - | - |
| Urease | - | - | - | - | - | - | - | - |
| Hydrolysis of: |  |  |  |  |  |  |  |  |
| L-Arginine | + | + | + | + | + | + | + | **-*** |
| L-Lysine | - | - | - | - | - | - | - | - |
| L-Ornithine | - | - | - | - | - | - | - | - |
| L-Tryptophane | - | - | - | - | - | - | - | - |
| Gelatinase | + | + | + | + | + | + | + | + |
| Assimilation of: |  |  |  |  |  |  |  |  |
| D-Glucose | + | + | + | + | + | + | + | + |
| D-Mannitol | + | + | + | + | + | + | + | + |
| Inositol | - | - | - | - | - | - | - | - |
| D-Sorbitol | - | - | - | - | - | - | - | - |
| L-Rhamnose | - | - | - | - | - | - | - | - |
| D-Sucrose | - | - | - | - | - | - | - | - |
| D-Melibiose | - | - | - | - | - | - | - | - |
| Amygdalin | + | + | + | + | + | + | + | + |
| L-Arabinose | - | - | - | - | - | - | - | - |
| Oxidase | + | + | + | + | + | + | + | + |
| + positive, -negative, * different reaction compared to wild-type and other mutant strains | | | | | | | | |

**Table S6.** Biochemical profiles of wild-type and mutants of *A. salmonicida* using the API 20NE

| **Enzyme Assayed for** | **J223** | ***∆ribA*** | ***∆ribAB*** | ***∆ribB*** | ***∆ribE1*** | ***∆ribE2*** | ***∆ribN*** | ***∆ribA*-*∆ribE1*** |
| --- | --- | --- | --- | --- | --- | --- | --- | --- |
| Reduction of nitrates to nitrites | + | + | + | + | + | + | + | + |
| Indole production | - | - | - | - | - | - | - | - |
| Glucose fermentation | + | + | + | + | + | + | + | + |
| Urease | - | - | - | - | - | - | - | - |
| β-Galactosidase | - | - | - | - | - | - | - | - |
| Hydrolysis of: |  |  |  |  |  |  |  |  |
| Arginine | + | + | + | + | + | + | + | **-*** |
| Esculin | + | + | + | + | + | + | + | + |
| Gelatin | + | + | + | + | + | + | + | + |
| ­Assimilation of: |  |  |  |  |  |  |  |  |
| D-glucose | + | + | + | + | + | + | + | + |
| L-arabinose | - | - | - | - | - | - | - | - |
| D-mannose | + | + | + | + | + | + | + | + |
| D-mannitol | + | + | + | + | + | + | + | + |
| N-acetyl-glucosamine | + | + | + | + | + | + | + | + |
| D-maltose | + | + | + | + | + | + | + | + |
| Potassium gluconate | - | - | - | - | - | - | - | - |
| Capric acid | - | - | - | - | - | - | - | - |
| Adipic acid | - | - | - | - | - | - | - | - |
| Malic acid | + | + | + | + | + | + | + | + |
| Trisodium citrate | - | - | - | - | - | - | - | - |
| Phenylacetic acid | - | - | - | - | - | - | - | - |
| + positive, -negative, * different reaction compared to wild-type and other mutant strains | | | | | | | | |

**Table S7.** Enzymatic profiles of wild-type and mutants of *A. salmonicida* using the API-ZYM

| **Enzyme Assayed for** | **J223** | ***∆ribA*** | ***∆ribAB*** | ***∆ribB*** | ***∆ribE1*** | ***∆ribE2*** | ***∆ribN*** | ***∆ribA*-*∆ribE1*** |
| --- | --- | --- | --- | --- | --- | --- | --- | --- |
| Alkaline phosphatase | + | + | + | + | + | + | + | + |
| Esterase (C_4_) | + | + | + | + | + | + | + | + |
| Esterase lipase (C_8_) | + | + | + | + | + | + | + | + |
| Lipase (C_14_) | + | + | + | + | + | + | + | + |
| Leucine arylamidase | + | + | + | + | + | + | + | + |
| Valine arylamidase | ± | ± | ± | ± | ± | ± | ± | ± |
| Cystine arylamidase | - | - | - | - | - | - | - | - |
| Trypsin | - | - | - | - | - | - | - | - |
| α-Chymotrypsin | - | - | - | - | - | - | - | - |
| Acid Phosphatase | + | + | + | + | + | + | + | + |
| Naphthol-AS-BI-Phosphohydrolase | + | + | + | **-*** | + | + | + | + |
| α-galactosidase | - | - | - | - | - | - | - | - |
| β-galactosidase | - | - | - | - | - | - | - | - |
| β-glucoronidase | - | - | - | - | - | - | - | - |
| α-glucosidase | - | - | - | - | - | - | - | - |
| β-glucosidase | + | + | + | + | + | + | + | + |
| N-acetyl-β-glucosaminidase | + | + | + | + | + | + | + | + |
| α-mannosidase | - | - | - | - | - | - | - | - |
| α-fucosidase | - | - | - | - | - | - | - | - |
| + positive, -negative, ± weak positive, * different reaction compared to wild-type and other mutant strains | | | | | | | | |
