# Supplementary figures and images for: Role of riboflavin biosynthesis gene duplication and transporter in Aeromonas salmonicida virulence in marine teleost fish
Source: Virulence. 2023 Mar 9;14(1):2187025. doi: 10.1080/21505594.2023.2187025 (PMC10012899; doi:10.1080/21505594.2023.2187025)

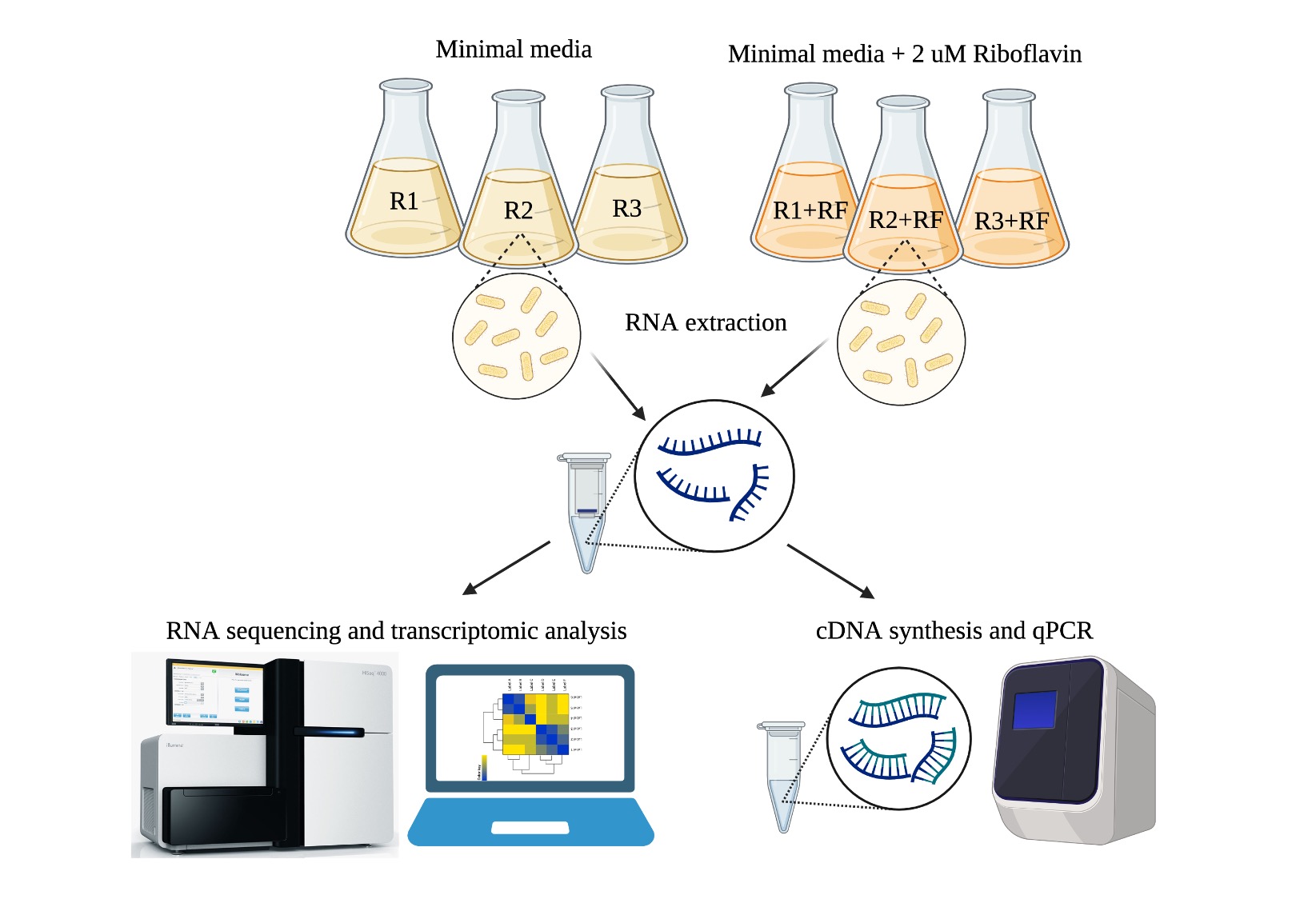

Supplement: Supplemental Material [file KVIR_A_2187025_SM7317.zip › Fig S1.jpg]

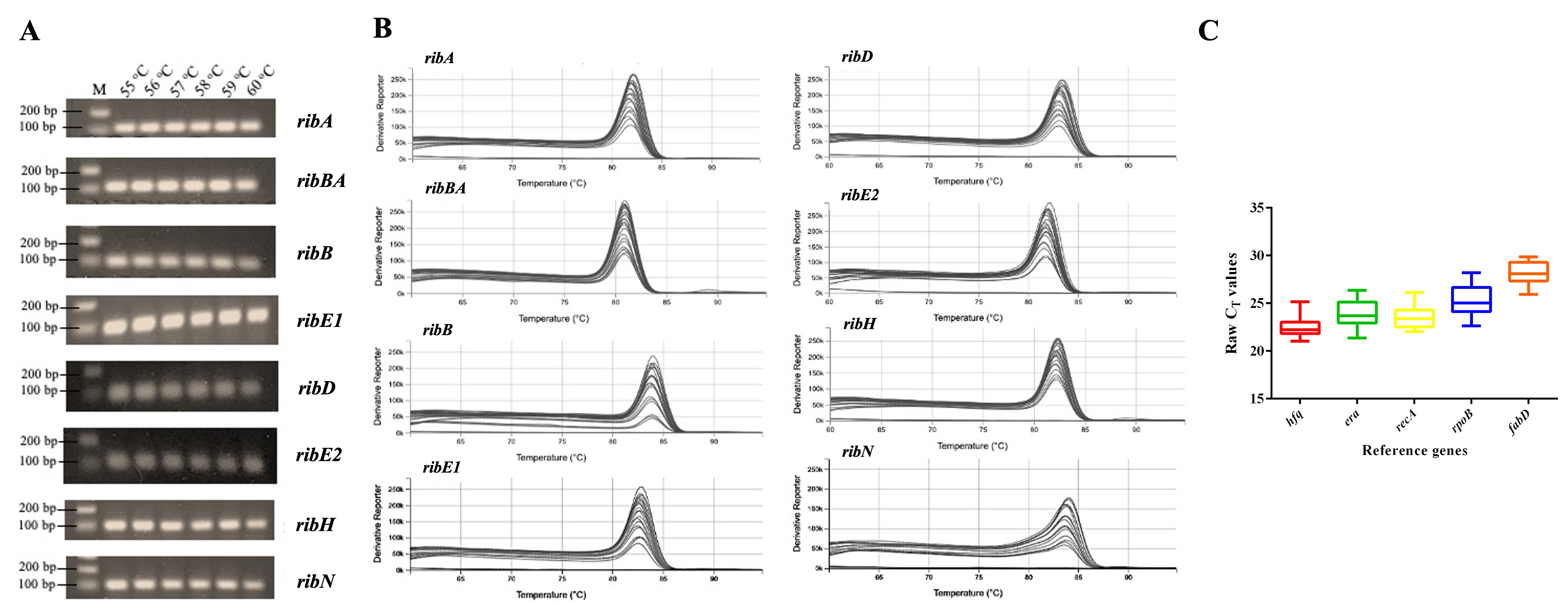

Supplement: Supplemental Material [file KVIR_A_2187025_SM7317.zip › Fig S2.jpg]

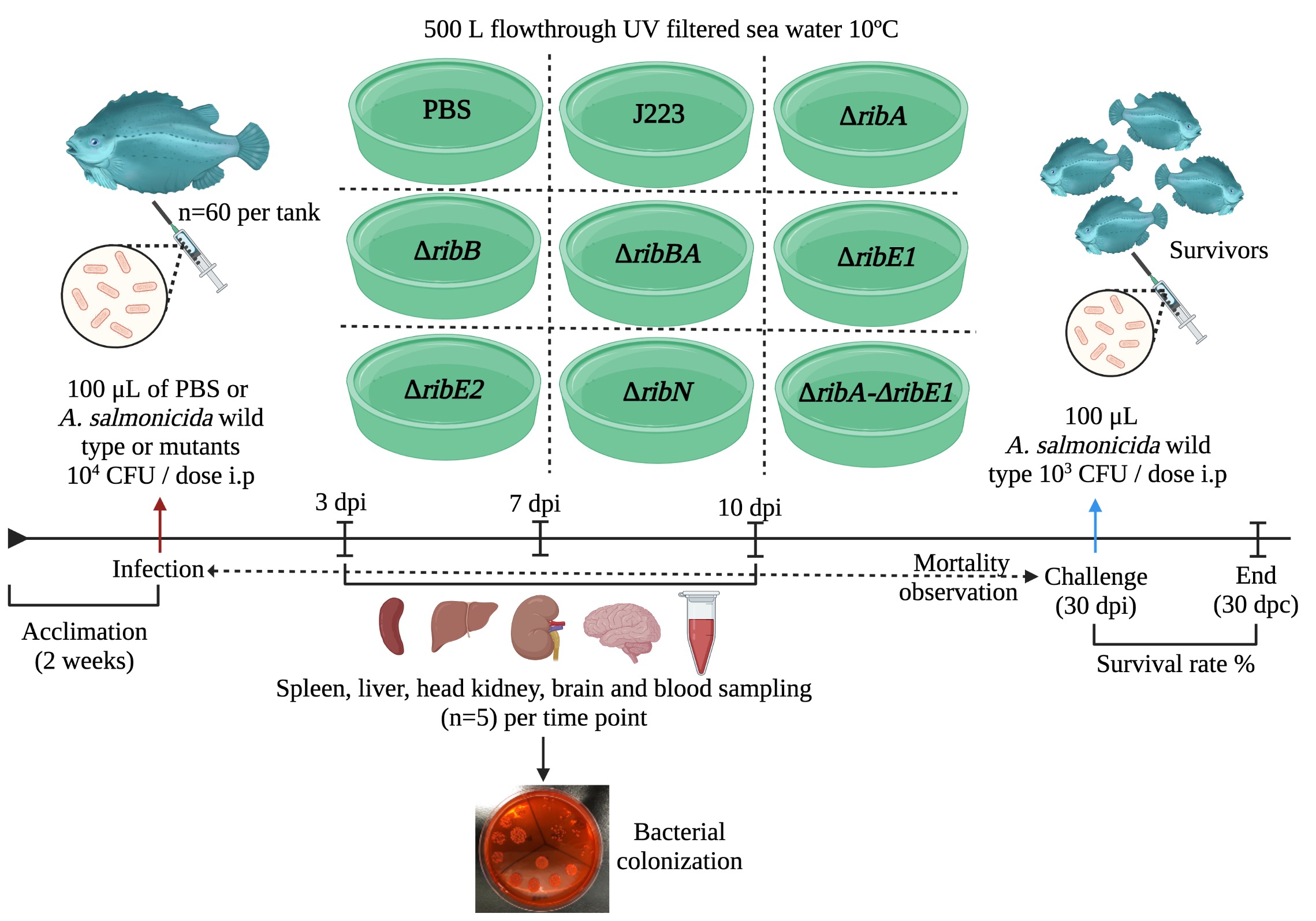

Supplement: Supplemental Material [file KVIR_A_2187025_SM7317.zip › Fig S3.jpg]

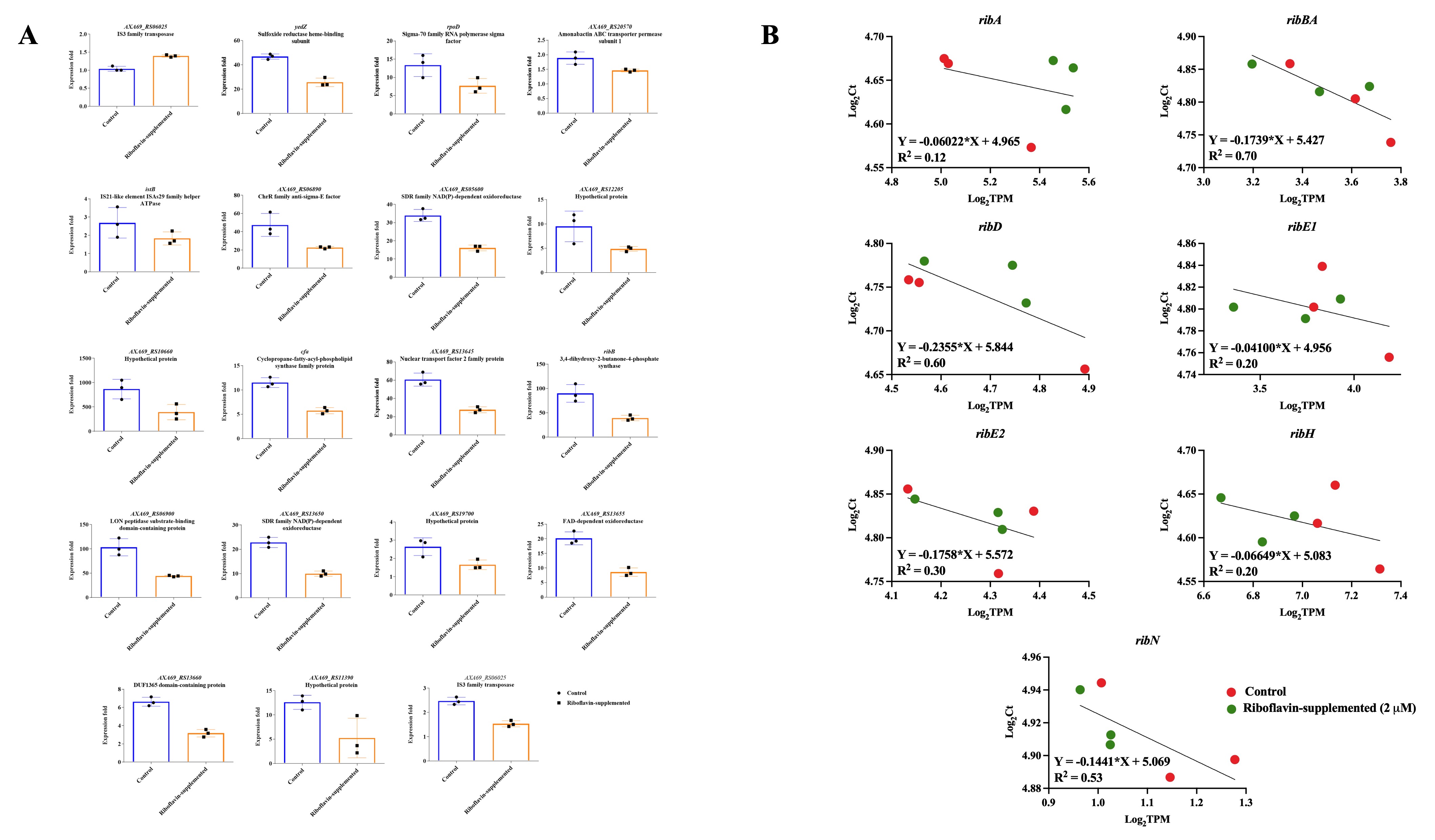

Supplement: Supplemental Material [file KVIR_A_2187025_SM7317.zip › Fig S4.jpg]

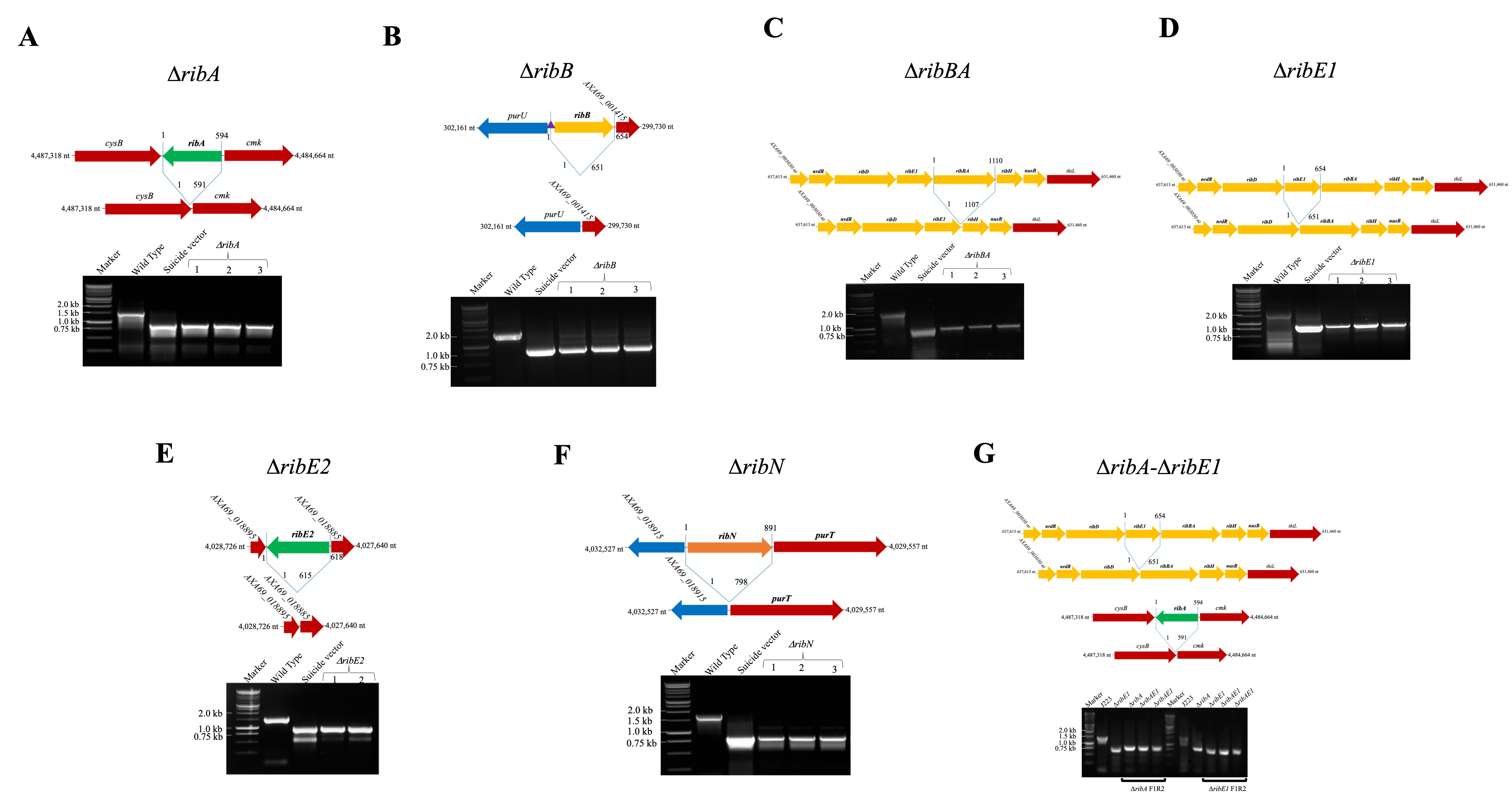

Supplement: Supplemental Material [file KVIR_A_2187025_SM7317.zip › Fig. S5.jpg]
